# Identification and analysis of oxidative stress-related genes in endometriosis

**DOI:** 10.3389/fimmu.2025.1515490

**Published:** 2025-03-07

**Authors:** Chengmao Xie, Chang Lu, Na Lv, Weimin Kong, Yong Liu

**Affiliations:** Department of Gynecology, Beijing Obstetrics and Gynecology Hospital, Capital Medical University, Beijing Maternal and Child Health Care Hospital, Beijing, China

**Keywords:** endometriosis, ectopic endometrium, eutopic endometrium, oxidative stress, machine learning, immune cells, lncRNA

## Abstract

**Background:**

Early diagnosis and treatment of endometriosis (EM) remain challenging because of the lack of knowledge about EM development. While oxidative stress (OS) has been associated with EM, the link is unclear. We explored OS-related genes (OSRGs) and their role in EM pathogenesis.

**Material and methods:**

We combined two ectopic endometrium (EC) and eutopic endometrium (EU) datasets (GSE11691 and GSE25628) into a dataset for analysis. Bioinformatic analyses were used to identify differentially expressed genes (DEGs), OS-related genes (OSRGs), enriched pathways, competitive endogenous RNA network, and immune cell infiltration. Finally, real time-quantitative polymerase chain reaction (RT-qPCR) and Western blot (WB) were used to validate the expression of key OSRGs in clinical patient samples.

**Results:**

Bioinformatic analysis identified 459 DEGs between EC and EU samples, including 67 OSRGs. A ceRNA network was established, encompassing 28 DE-OSRGs, 32 miRNAs, and 53 lncRNAs. Four key OSRGs (*CYP17A1*, *NR3C1*, *ENO2*, and *NGF*) were selected from protein-protein interaction network analysis. The RT-qPCR and WB analysis showed that these genes’ abnormal changes in RNA and protein levels were consistent with data in public databases. Weighted gene co-expression network analysis identified three immune-related OSRGs (*CYP17A1, NR3C1,* and *NGF*) and 20 lncRNAs that may regulate *NR3C1* through 10 miRNAs.

**Conclusion:**

The key OSRGs may function via multilayered networks in EM. We provide insights into EM and underscore the potential significance of OSRGs and the immune environment for diagnostic and prognosis evaluation.

## 1 Introduction

Endometriosis (EM) is a chronic disease characterized by the development of lesions, starting as speckled spots on the ovary and progressing into deep infiltrating cysts filled with brown fluid. These cysts, along with endometrioid tissue, are located outside the uterine cavity (1, 2). Ectopic lesions predominantly consist of interstitial cells and glands sensitive to hormonal fluctuations; these lesions are commonly found in the pelvic region, particularly affecting the ovaries, pelvic peritoneum, uterosacral ligament, and fallopian tubes (3). Individuals with EM commonly experience a range of symptoms, including pelvic pain, irregular menstrual bleeding, dysmenorrhea, difficulty in sexual intercourse, abdominal and pelvic pain during non-menstrual periods, dysuria, and gastrointestinal disturbances such as constipation and diarrhea. Approximately 70% of EM patients experience chronic pelvic pain, and 30%–50% encounter infertility (4). The increased surgical intervention decreased quality of life, and high prevalence of assisted reproductive technology caused by EM have led to a significant increase in social costs (5). While medical treatment can alleviate symptoms in up to 50–80% of cases, approximately 20% of patients still have symptoms (6–8). More effective treatments are lacking, mainly because the mechanism underlying the pathogenesis of EM remains unclear. Consequently, identifying novel biomarkers is imperative to deepen understanding of etiology and molecular underpinnings of EM and to identify novel targets for its clinical diagnosis and therapeutic intervention.

Ectopic endometrium (EC) undergoes three stages of “adhesion, invasion, and angiogenesis” to ultimately lead to the occurrence of EM (9). The related cytokines, extracellular matrix degradation, and angiogenesis processes are increased by oxidative stress (OS), leading to the occurrence of EM (10). OS is defined as a stress state in which the balance between oxidation and antioxidant systems is disrupted. A large number of oxygen-free radicals accumulate that exceeds the body’s ability to clear oxygen free radicals, leading to inflammatory infiltration of neutrophils and the production of pain-inducing factors such as macrophages, enhanced pain-related protease activity, promoted lipid peroxidation, and pelvic pain (11). Excessive oxygen free radicals can extensively oxidize and damage DNA, lipids, or proteins in cells, directly disrupting the cellular structure and physiological functions. They also serve as secondary messengers, indirectly leading to the occurrence and development of various diseases, such as EM, by activating related factors and signaling pathways (12, 13). Excessive reactive oxygen species (ROS) activation of the NF-κB signaling pathway upregulates expression of adhesion factor-1 (ICAM-1), alters the morphology of peritoneal epithelial cells, provides adhesion sites for ectopic endometrium, and upregulates interleukin-8 (IL-8) and tumor necrosis factor-β (TGF-β) inflammatory factors (9, 14, 15). Inflammatory factors and highly expressed superoxide dismutase (SOD2), which counteracts excessive oxygen-free radical stress, can mediate the Ras/Raf/MEK/ERK signaling pathway to stimulate extracellular regulatory protein kinase (ERK1/2) phosphorylation and upregulate levels of MMP-2 and MMP-9, accelerating extracellular matrix degradation and enhancing the proliferation, invasion, and migration of EM ectopic endometrial cells (16, 17). ROS also activates the MAPK/ERK signaling pathway to regulate c-Fos and c-Jun factors, inhibit cell apoptosis, and promote ectopic endometrial cell proliferation in EM (18).

While the role of OS in the pathogenesis of EM has been investigated, the molecular mechanism underlying EM pathogenesis is still limited and requires further research. Here, we performed a comparative transcriptome analysis of ectopic endometrium (EC) and eutopic endometrium (EU) samples to identify OS-related genes (OSRGs) and immune cells that are critical in EM development. Our studies results provide new insights into the pathogenesis of EM and underscore the potential significance of OSRGs and the immune environment for diagnostic values and prognosis evaluation in EM.

## 2 Materials and methods

### 2.1 Integrated bioinformatics analysis

#### 2.1.1 Data sources

We downloaded four EM-related transcriptome datasets (GSE11691, GSE25628, GSE105764 and GSE105765) from GEO database (19–22). The nine EU samples and nine EC samples in the GSE11691 dataset and the seven EC samples and nine EU samples in the GSE25628 dataset were used as the combined dataset for the main analysis (20). The “ComBat” algorithm was applied to reduce batch effects caused by non-biotechnological biases in the expression profile of composite datasets. Principal component analysis (PCA) was performed on data before and after batch calibration. The GSE105764 and GSE105765 datasets were used to construct the competitive endogenous RNA (ceRNA) network, with each dataset containing eight EC samples and eight EU samples (22). The 1,150 OSRGs were derived from the GeneCards database (https://www.genecards.org/) with the keyword “oxidative stress related gene” by setting the criterion as “relevance score ≥ 7” (23).

**Figure 1:**
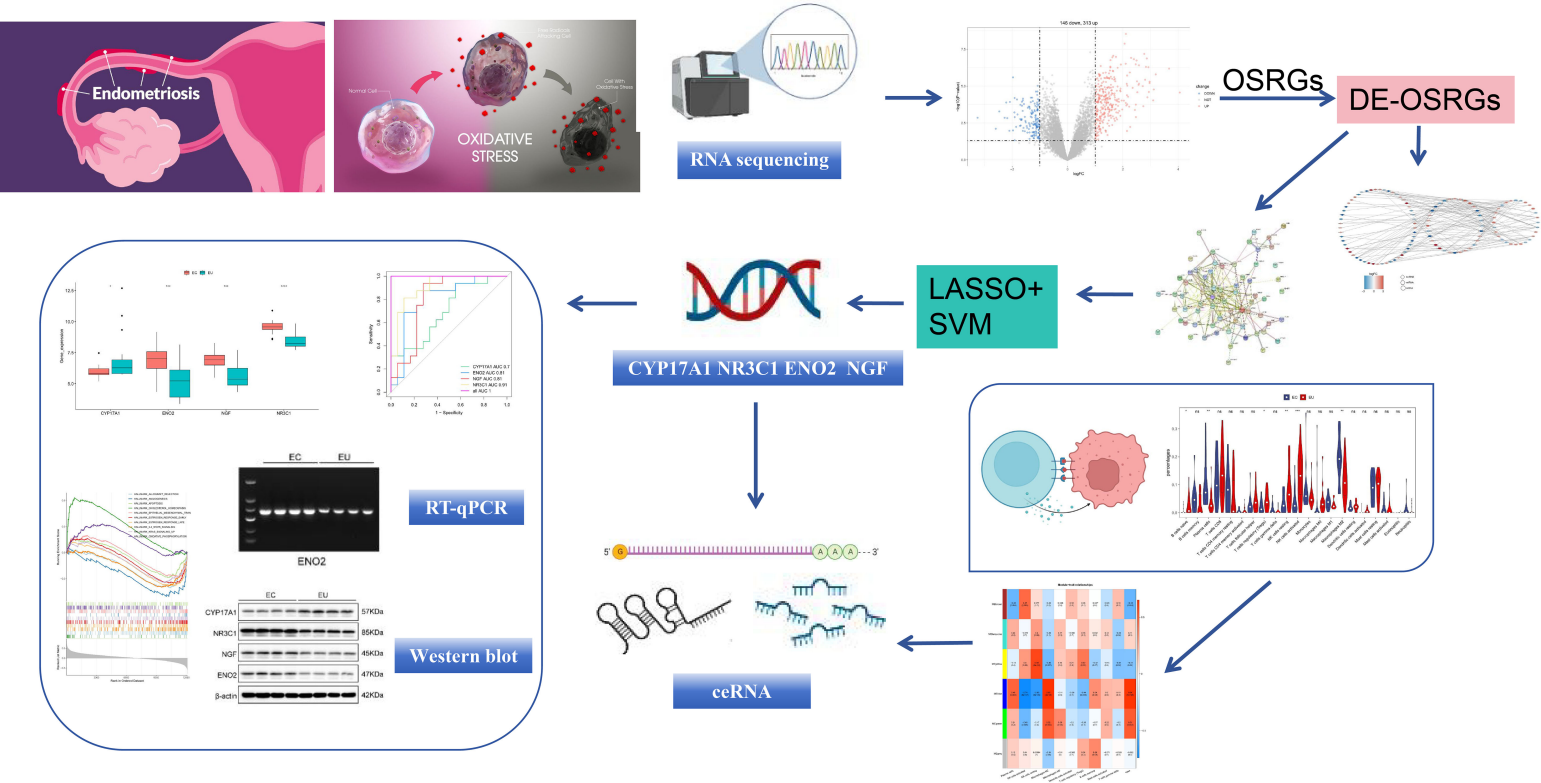
Schematic diagram of the overall study design.

The overall study design is shown in **Figure 1**.

#### 2.1.2 Identification of differentially expressed OSRGs

Differentially expressed genes (DEGs) from EC and EU samples in the combined dataset were determined using ‘limma’ package (v 3.46.0) (24, 25). *P* < 0.05 and |Log_2_FC|>1 were applied as the cut-off criteria. DE-OSRGs were acquired by overlapping DEGs and OSRGs by the ‘ggplot2’ package (v 3.3.2) (26).

#### 2.1.3 Construction of the ceRNA regulation network

To investigate the molecular regulatory mechanisms of EM, differentially expressed miRNAs (DE-miRNAs) in the GSE105765 dataset were screened out via DESeq2 package (v 1.30.1) with *P* < 0.05 and |Log_2_FC|>1 (27). We also used the DESeq2 package to screen out differentially expressed lncRNAs (DE-lncRNAs) in the GSE105764 dataset with *P* < 0.05 and |Log_2_FC|>1. The starbase database (https://rnasysu.com/encori/) was used to predict the miRNAs that interact with DE-OSRGs. We determined the intersection of the miRNA predictions across DE-miRNAs and the starbase database. The lncRNAs corresponding to miRNAs were predicted in the starBase database. We then identified DE-lncRNAs that overlapped with the predicted results from starBase database to obtain the final lncRNAs.

#### 2.1.4 Functional enrichment analysis of DE-OSRGs and PPI network

To explore potential biological functions and signaling pathways associated with DE-OSRGs, Gene Ontology (GO) and Kyoto Encyclopedia of Genes and Genomes (KEGG) enrichment analysis of DE-OSRGs were completed via ‘clusterProfiler’ package (v 4.0.2). *P <*0.05 and count ≥ 1 were set as the threshold value (28). To explore the interactions between proteins coded by the detected DE-OSRGs, we used the search tool for the retrieval of interacting genes (STRING) database (https://string-db.org/), along with Cytoscape software (3.8.2) (29). This approach allowed us to examine the relationships among DE-OSRGs and assemble a protein-protein interaction (PPI) network. We selected DE-OSRGs with connectivity >5 for subsequent analysis, and the top 20 DE-OSRGs were shown.

#### 2.1.5 Development of machine learning diagnostic models

For further exploration of key OSRGs, based on DE-OSRGs with connectivity >5 in PPI network, we performed least absolute selection and shrinkage operator (LASSO) Cox analysis via ‘glmnet’ package (v 4.1-4) and support vector machine recursive feature elimination (SVM-RFE) algorithm via ‘care’ package (v 6.0-92). The key OSRGs were obtained from the overlap of the two machine learning results (30, 31). To assess the diagnostic performance of key OSRGs, receiver operating characteristic (ROC) curves were plotted to calculate the area under the curve (AUC) for the combined dataset using the ‘pROC’ package (v 1.17.0.1) (32). The expression levels of key OSRGs were shown in the combined dataset.

#### 2.1.6 Gene set enrichment analysis

To explore the biological roles and pathways linked to key OSRGs, GSEA of each key OSRG was executed by the ClusterProfiler package based on the correlation between key OSRGs and other genes. The threshold for enrichment significance was adj. *P* < 0.05, and the reference gene set was the KEGG gene set (33).

#### 2.1.7 Immune infiltration analysis

To evaluate the immune cell composition within the immune landscape of EM patients, the CIBERSORT algorithm was applied to estimate the percentages of 22 immune cell types in the combined dataset. Violin plots were created to display the variations in immune cell infiltration between EC and EU samples (34, 35).

#### 2.1.8 Weighted gene co-expression network analysis

To further identify immune-related module genes, WGCNA (version 1.70.3) was used (36). A cluster analysis was conducted to determine whether the exclusion of outlier samples was necessary to ensure accuracy of subsequent analyses. A soft threshold (β) was selected to construct the co-expression network. Genes were compared for similarity according to their proximity, and a phylogenetic tree of the genes was created. The dynamic tree-cutting algorithm was used to partition the modules, with a minimum requirement of 300 genes per module. Gene modules with a stronger correlation with differential immune cells were used as the immune-related module for further analysis (37).

#### 2.1.9 Analysis of the ceRNA regulation network of key immune-related OSRGs

Key immune-related OSRGs were identified by intersecting the obtained immune-related module genes with key OSRGs. A ceRNA network was then constructed via key immune-related OSRGs.

### 2.2 Clinical samples

This study included 30 patients diagnosed with EM from Beijing Obstetrics and Gynecology Hospital that met the following inclusion criteria: body mass index of 19–25 kg/m^2^, no operative contraindication, non-vegetarian patients, and age between 20 and 45 years. The exclusion criteria were diabetes and other endocrine diseases and cardiopulmonary, liver, and serious gastrointestinal diseases. EC and EU tissues were collected from the same patient. All tissue samples were taken from tissues discarded during surgery. Samples were obtained after approval from our hospital’s ethics review committee. The samples were then snap-frozen in liquid nitrogen and stored for further analysis.

### 2.3 cDNA synthesis and reverse transcription quantitative polymerase chain reaction

RNA isolation and reverse transcription quantitative polymerase chain reaction (RT-qPCR) were performed as described previously (38). PCR reactions were conducted using 0.4 µM of forward and reverse primers, 1 µL of Light Cycler DNA Master SYBR Green I (10× concentrate, Roche), and 3 mM of MgCl_2_. To ensure full denaturation of cDNA prior to amplification, an initial step of denaturation was carried out at 95°C for 1 min and 15 sec to activate FastStart DNA polymerase. The reaction continued with desaturation at 95°C for 15 sec, annealing at 62°C for 10 sec, and extension for 40 cycles at 72°C for 25 seconds to amplify target genes and the endogenous control gene. We analyzed the amplification product and performed a melting curve analysis to check the integrity of the amplification after the last cycle. Data analysis was performed with LightCycler Relative Quantification Software, employing the second derivative max method for calculation. The primers used for RT-PCR analysis are as follows: *CYP17A1*, sense 5′-TTCAGCCGCACACCAACTAT-3′ and anti-sense 5′-GGACAG GGGCTGTG AGTTAC-3′; *ENO2*, sense 5′-TCGCTTTGCCGGACATAACT-3′ and anti-sense 5′-GACACAT CGTTCCCCCAAGT-3′; *NR3C1*, sense 5′-AGGAATAGAAACAGAAAGAGGTTGA -3′ and anti-sense 5′-ACTGGGGCTTGACAAAACCA-3′; and *NGF*, sense 5′-GCGCAGCGAGTT TTGGC-3′ and anti-sense 5′-GGATGGGATGATGACCGCTT-3′. The relative quantitation values were calculated using the ΔΔCt method. By subtracting the Ct value of the reference genes GAPDH/actin from the Ct value of the gene under investigation, the ΔCt value was derived. This was done to transform the output of PCR into risk scores. The ΔCt values of all samples were then used to calculate the average ΔCt value. Next, the difference between the ΔCt value and its average value (ΔΔCt) was obtained. Finally, -ΔΔCt values were used to calculate the relative expression level of the target gene through two exponentiations. The relative expression level is an alternative to the risk-scoring formula. After qRT-PCR, we verified the mRNA specificity of the key OSRGs with agarose gel electrophoresis. All experiments were performed at least three times.

### 2.4 Western blot analysis

Protein lysate preparation and western blotting analysis were performed as previously described (38). Protein extraction was performed using the ProteoExtract Native Membrane Protein Extraction Kit (M-PEK; Calbiochem, Darmstadt, Germany). The Quick Start Bradford protein assay (Thermo Fisher Scientific, Waltham, MA, USA) was used to determine protein concentration. We conducted SDS-PAGE with approximately 20 µg of protein sample loaded onto 7% triethyl acetate gels at 120 V for 2 h. Samples were not heated before electrophoresis to maintain the integrity of the target protein. Proteins were transferred to an Immobilization NC transfer membrane under 300 mA for 90 min. The membrane was blocked in 5% skim milk powder containing 0.1% Tween 20 (TBST) Tris buffer saline for 2 h and incubated overnight at 4°C with the rabbit monoclonal anti-Cytochrome P450 17A1/CYP17A1 antibody (ab134910; Abcam, Cambridge, UK), rabbit monoclonal anti-ENO2 antibody (ab133309; Abcam, Cambridge, UK), rabbit monoclonal anti-NGF antibody (ab52987; Abcam, Cambridge, UK), and rabbit monoclonal anti-NR3C1 antibody (ab3671; Abcam, Cambridge, UK) (1:1000 in TBST). The membrane was washed three times with TBST for 10 min each. Next, the membrane was incubated with the secondary antibody (1:5000 in TBST) conjugated with mouse anti-rabbit IgG horseradish peroxidase (HRP) at room temperature (RT) for 45 min. After washing the membrane three times for 10 minutes in TBST, the signal was recorded by digital imaging using ChemiDocTM XRS+and Image Lab™ Software (BIO-RAD, Hercules, CA, USA). *β*-actin was used as an internal control. We used ImageJ for density measurement and quantitative analysis (value=absorbance of the target band/β-actin absorbance). All experiments were performed at least three times.

### 2.5 Statistical analysis

All analyses were executed in R language (v 4.2.3). Differences between groups were analyzed by the Wilcoxon test. *P* < 0.05 was considered statistically significant.

## 3 Results

### 3.1 Identification of differentially expressed OSRGs from the combined dataset of EC and EU samples

**Figure 2:**
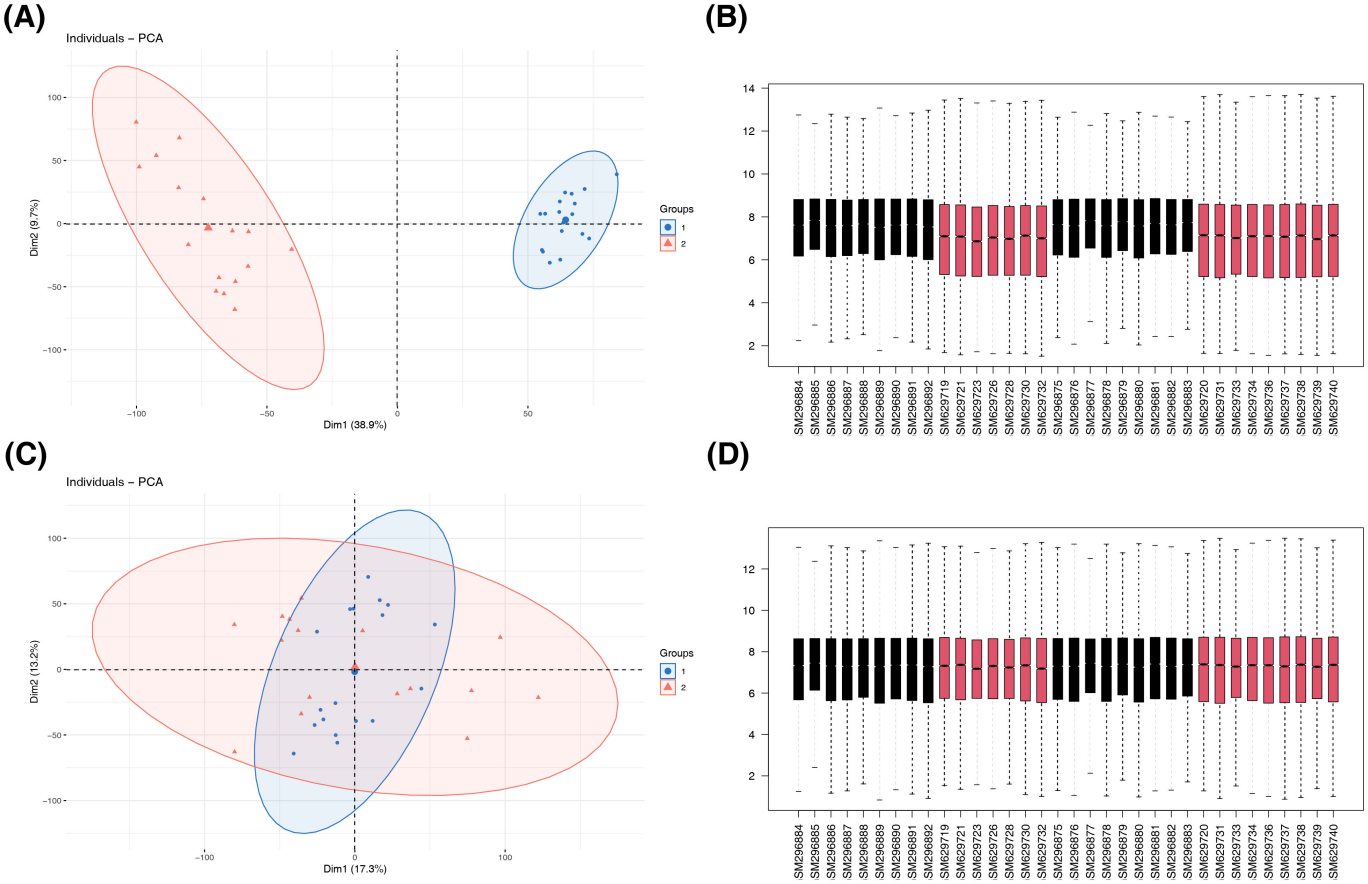
Visualization of the data processing for GSE11691 and GSE25628, highlighting the effects of background adjustment and quantile normalization. **(A)** The principal component analysis (PCA) results before correction. **(B)** The box plots before normalization. **(C)** The PCA results after correction. **(D)** The box plots after normalization.

To identify the differentially expressed OSRGs involved in EM, we first integrated two sample datasets (GSE11691 and GSE25628). The Datasets GSE11691 and GSE25628 underwent background adjustment and quantile normalization prior to their merger. Subsequently, we eliminated batch effects, and gene expression profiles were generated from 34 samples, encompassing 18 EU and 16 EC across 12,403 genes. Visual assessments using box plots and PCA confirmed the effective mitigation of batch effects (**Figures 2A–D**).

**Figure 3:**
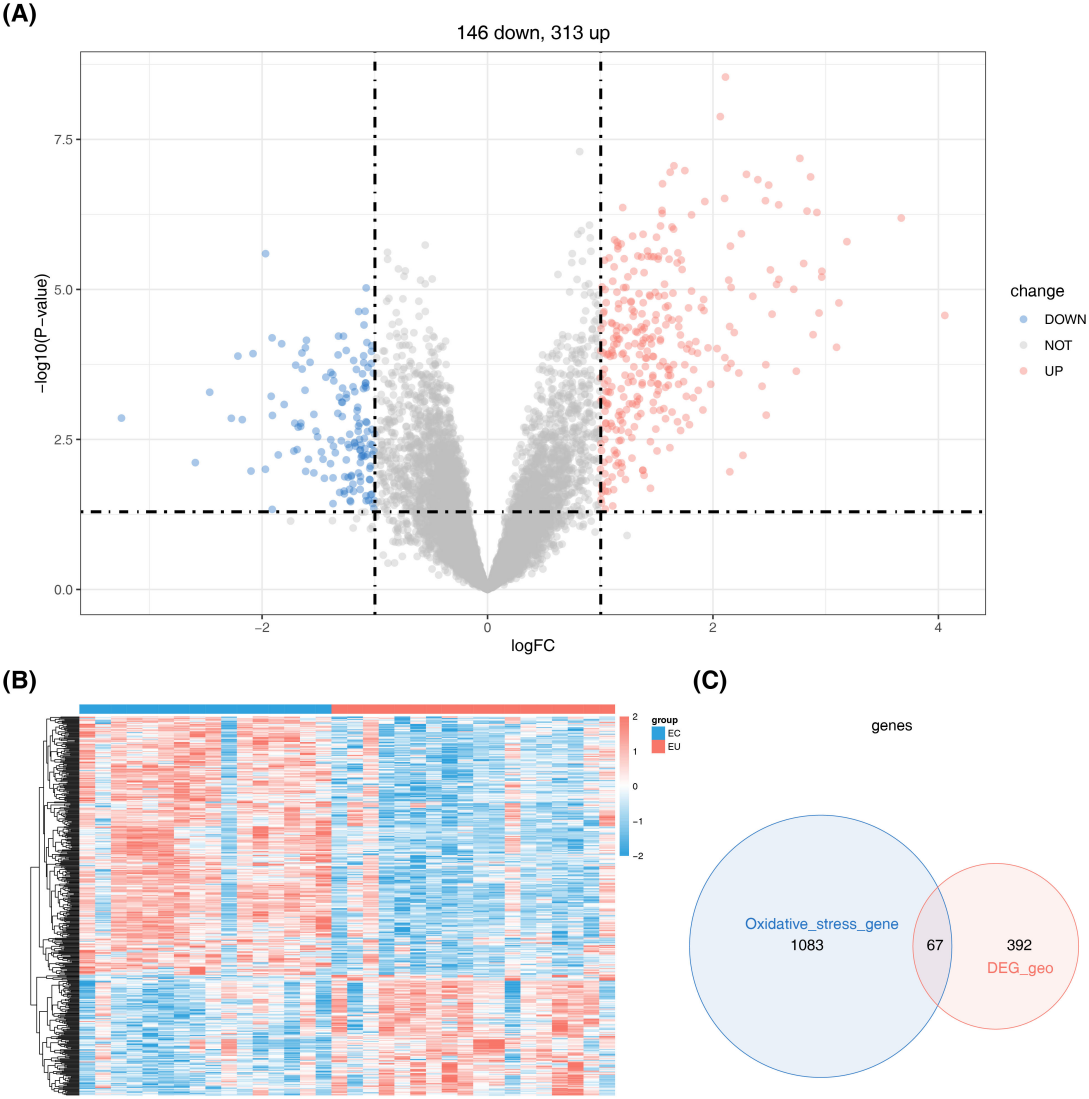
Identification of differentially expressed genes (DEGs) between ectopic endometrium (EC) and eutopic endometrium (EU) samples. **(A)** Volcano plot representing the distribution of DEGs. **(B)** Heat map detailing the expression patterns of the DEGs. **(C)** Venn diagram showing the intersection of 67 DE-OSRGs between DEGs and OSRGs.

Subsequent data analysis identified 459 DEGs, including 313 up-regulated and 146 down-regulated DEGs, from the combined EC and EU sample dataset. The volcano plot and heatmap showed the distribution of DEGs. (**Figures 3A, B**). By overlapping DEGs with OSRGs, we identified 67 differentially expressed OSRGs (DE-OSRGs) (**Figure 3C**).

### 3.2 Construction of a ceRNA network of DE-OSRGs

**Figure 4:**
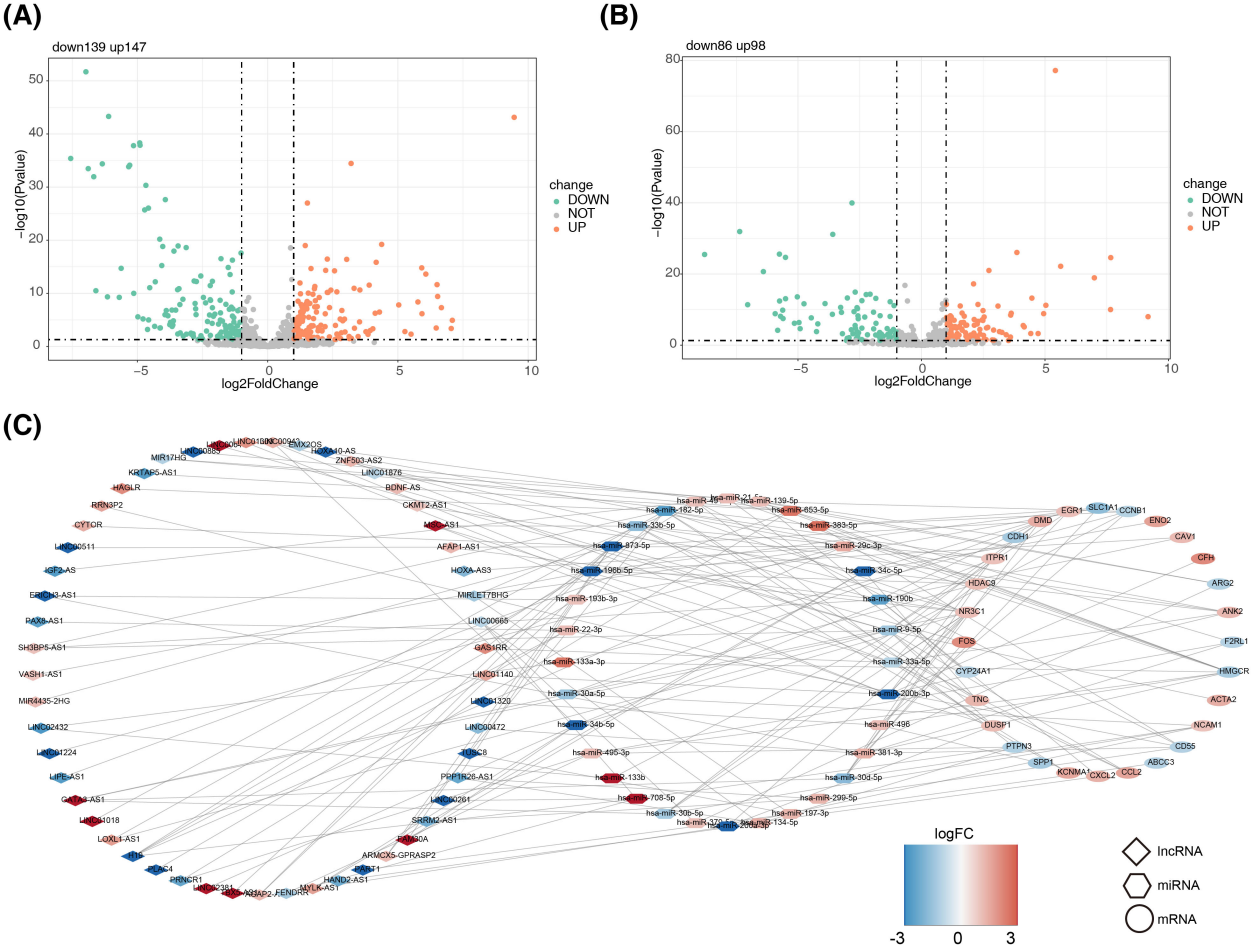
Construction of the competitive endogenous RNA (ceRNA) regulation network. **(A)** Volcano plot representing the distribution of differentially expressed miRNAs (DE-miRNAs). **(B)** Volcano plot representing the distribution of differentially expressed lncRNAs (DE-lncRNA). **(C)** ceRNA network encompassing mRNAs, miRNAs, and lncRNAs.

We identified 286 DE-miRNAs (147 up-regulated and 139 down-regulated) from the GSE105765 dataset (**Figure 4A**). In addition, we identified 184 DE-lncRNA (98 up-regulated and 86 down-regulated) from the GSE105764 dataset (**Figure 4B**). A ceRNA network was constructed by overlapping DE-miRNA and DE-lncRNAs with the predicted results from the starBase database. Specifically, the ceRNA network analysis revealed that 28 DE-OSRGs could interact with 32 miRNAs, which could, in turn, interact with 53 lncRNAs (**Figure 4C**).

### 3.3 Function enrichment analysis and PPI network of DE-OSRGs

**Figure 5:**
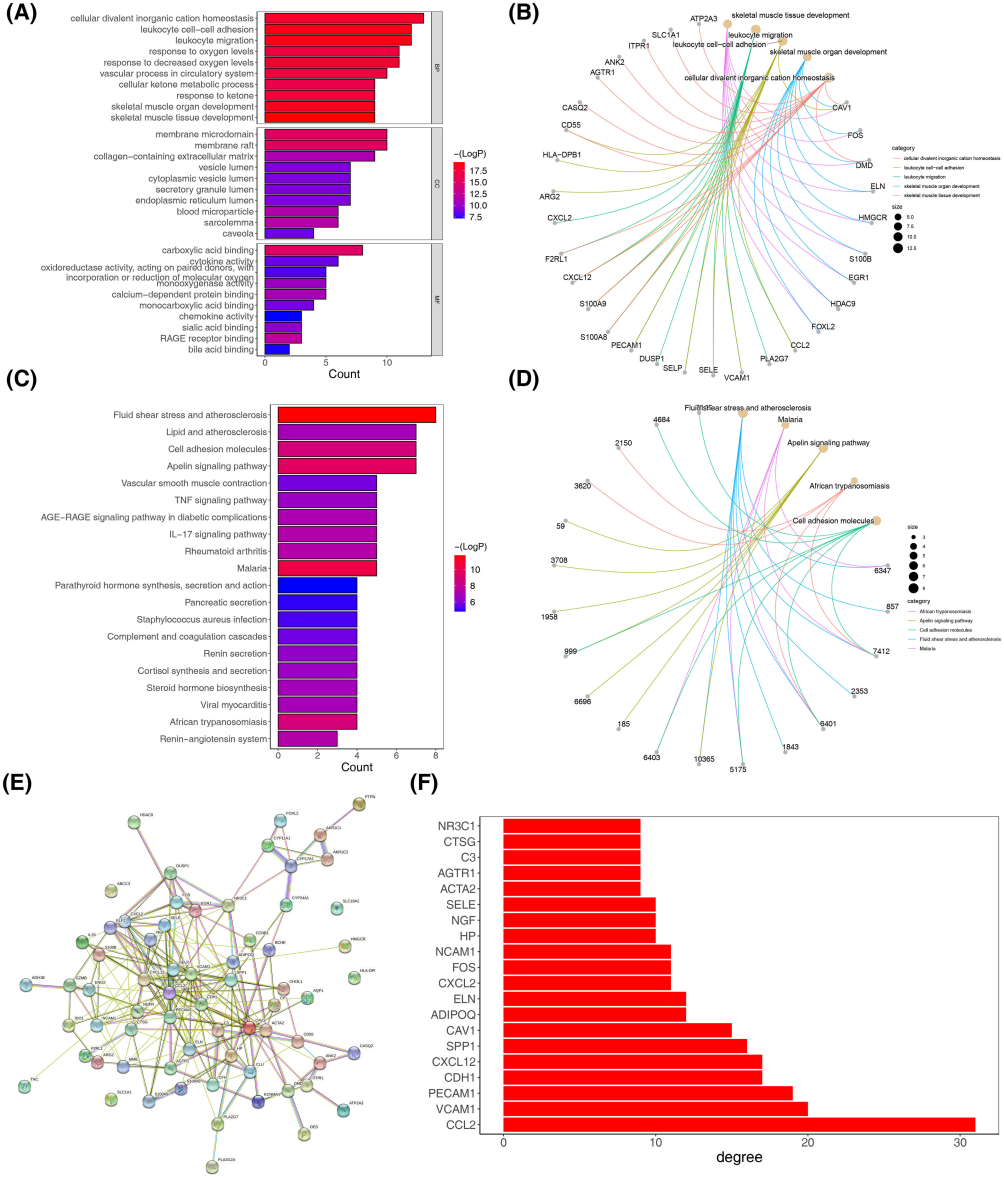
Function enrichment analysis and protein-protein interaction (PPI) network. **(A, B)** Gene Ontology (GO) of DE-OSRGs. **(C, D)** Kyoto Encyclopedia of Genes and Genomes (KEGG) of DE-OSRGs. **(E)** PPI network of DE-OSRGs. **(F)** The top 20 genes with the highest connectivity.

Following the previous analysis, DE-OSRGs were incorporated into the GO and KEGG analyses. DEGs were primarily associated with “cellular divalent inorganic cation homeostasis,” “leukocyte cell-cell adhesion,” “leukocyte migration,” “skeletal muscle organ development,” and “skeletal muscle tissue development” in GO entries (**Figures 5A, B**) and were mainly enriched in “African trypanosomiasis,” “apelin signaling pathway,” “cell adhesion molecules,” “fluid shear stress and atherosclerosis,” “malaria” and other KEGG pathways (**Figures 5C, D**). These analyses revealed that DE-OSRGs were associated with multiple biological functions and implicated disease-related pathways. To characterize the interaction of DE-OSRGs at the protein level, we established a PPI network containing 63 nodes and 220 edges; for instance, *CYP17A1* was associated with *FOXL2, CYP17A1, AKR1C2*, and *NR3C1* (**Figure 5E**). **Figure 5F** shows the top 20 genes with the highest connectivity.

### 3.4 Identification of key OSRGs

**Figure 6:**
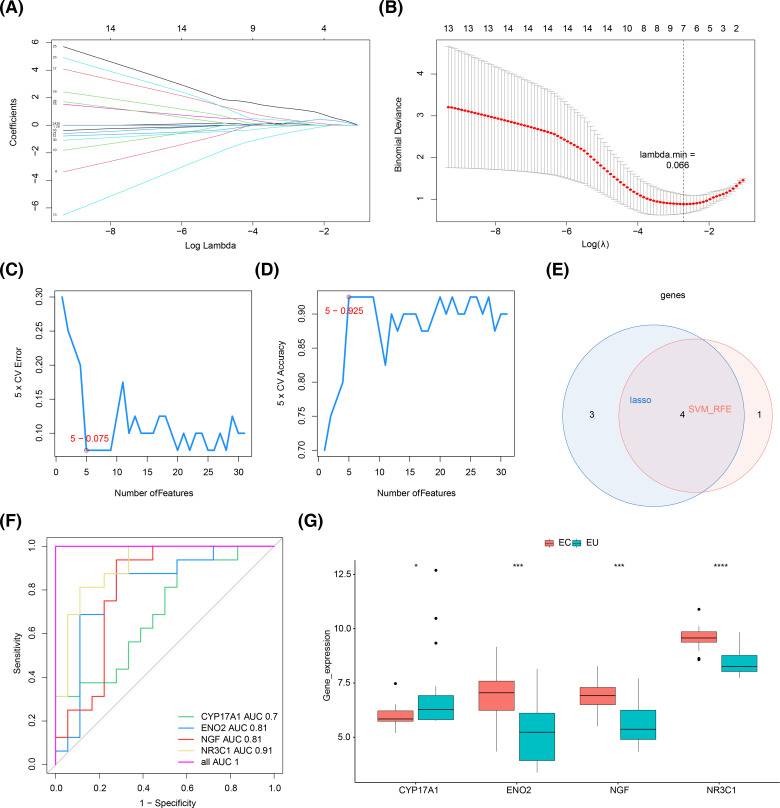
The development of the machine learning diagnostic models and key OSRGs. **(A, B)** Among the 31 DE-OSRGs, *ACTA2, CYP17A1, ENO2, NGF, NR3C1, SELP,* and *SPP1* were identified with a connectivity score greater than five by least absolute selection and shrinkage operator (LASSO) algorithm. **(C, D**) *CYP17A1, NR3C1, ENO2, NGF,* and *CCL2* were pinpointed via the Support vector machine recursive feature elimination (SVM-RFE) algorithm. **(E)** *CYP17A1, NR3C1, ENO2,* and *NGF* were obtained by overlapping both machine learning results as key OSRGs. **(F)** Receiver operating characteristic (ROC) curve of the four key OSRGs. **(G)** Expression levels of four key OSRGs were compared in the combined dataset. *P < 0.05, ***P < 0.001, ****P < 0.0001.

By least absolute selection and shrinkage operator (LASSO) algorithm, we identified seven genes (*ACTA2*, *CYP17A1*, *ENO2*, *NGF*, *NR3C1*, *SELP*, and *SPP1*) among the 31 DE-OSRGs with a connectivity score greater than 5 (Lambda_min_ = 0.066) (**Figures 6A, B**). Using the support vector machine recursive feature elimination (SVM-RFE) algorithm, five genes were screened out: *CYP17A1, NR3C1, ENO2, NGF*, and *CCL2* (**Figures 6, D**). Finally, four key OSRGs, including *CYP17A1*, *NR3C1*, *ENO2*, and *NGF* were identified (**Figure 6E**). ROC curves indicated that the AUC of key OSRGs was greater than 0.7 in the combined dataset, demonstrating decent diagnostic performance of these key OSRGs for EM (**Figure 6F**). We then compared the expression levels of four key OSRGs in the combined dataset. *NR3C1, ENO2*, and *NGF* showed upregulated expression, while *CYP17A1* showed down-regulated expression in EC compared with EU samples (*P* < 0.05) (**Figure 6G**).

### 3.5 Comparison and validation of four OSRGs at RNA and protein levels

**Figure 7:**
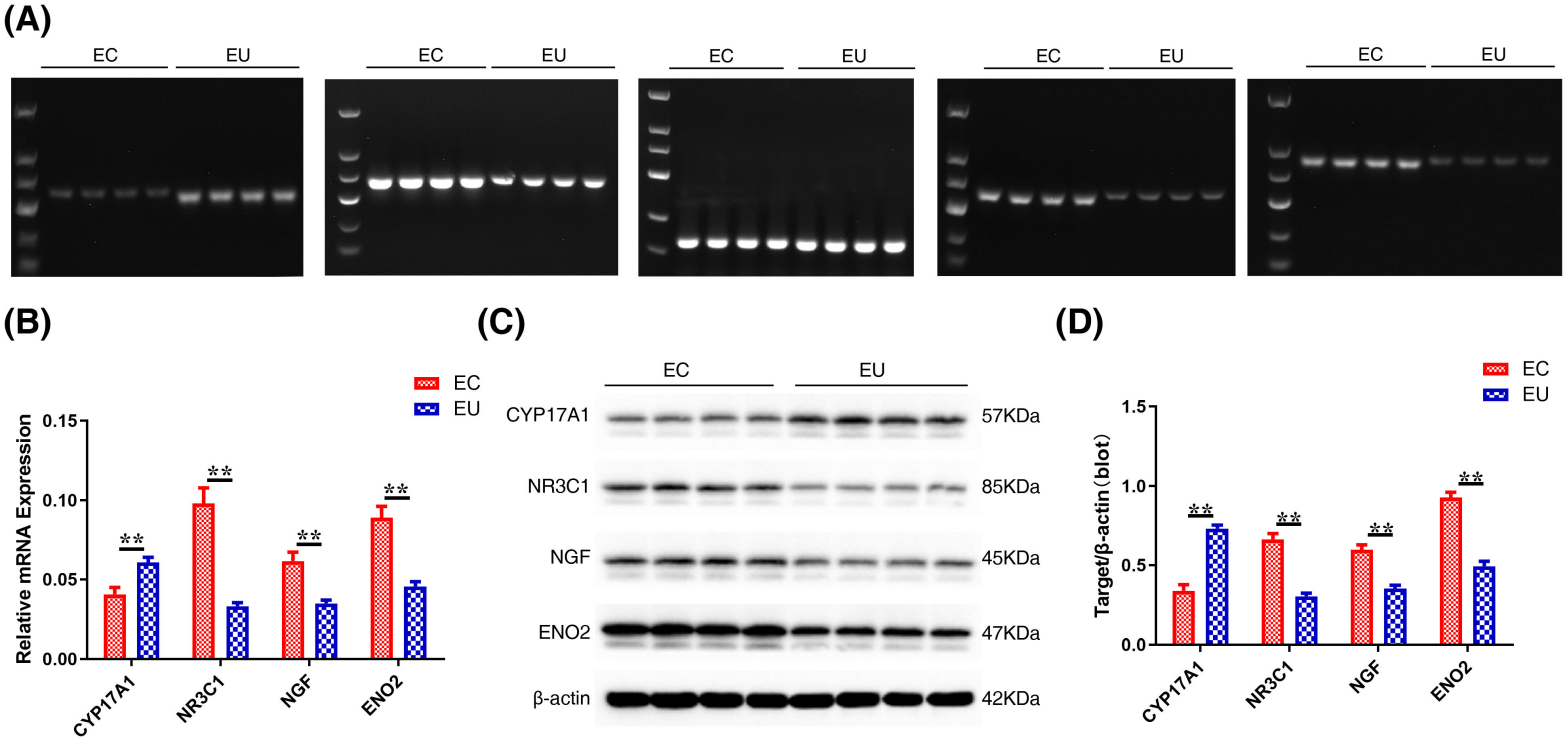
The mRNA and protein levels of key OSRGs. **(A)**The agarose gel electrophoresis of PCR amplification products of four key OSRGs in the indicated samples. *GAPDH* was used as an endogenous control. **(B)** RT-qPCR of four key OSRGs. **(C)** Western blot of four key OSRGs. **(D)** Quantitative analysis of OSRG proteins from panel **(C)**. β-actin was used as a loading control for western blot analysis. Data are presented as mean ± SD (n = 4). **P < 0.01.

To validate and compare the RNA levels between EC (n=15) and EU samples (n=15), PCR (**Figure 7A**) and RT-qPCR analysis (**Figure 7B**) were performed. The RNA expression of *NR3C1*, *NGF*, and *ENO2* in the EC samples was upregulated compared with EU samples in the PCR results (**Figure 7A**). RT-qPCR analysis demonstrated that the RNA expression of *NR3C1*, *NGF*, and *ENO2* in the EC samples was significantly higher than in the EU samples. Moreover, the RNA expression of *CYP17A1* was downregulated in EC samples compared with EU samples in both PCR and RT-qPCR results.

To validate and compare the protein levels between EC and EU samples, we performed western bot analysis of the same EC (n=15) and UC samples (n=15) used for RNA analysis. As shown in **Figures 7C, D**, NR3C1, NGF, and ENO2 protein levels were significantly upregulated, while CYP17A1 protein levels were significantly downregulated in EC samples compared with EU samples, indicating that the abnormal changes of NR3C1, NGF, ENO2, and CYP17A1 proteins in EC samples may occur through transcriptional regulation. Notably, our results were consistent with the microarray data (GSE11691 and GSE25628).

### 3.6 Identification of key OSRG-related signaling pathways

**Figure 8:**
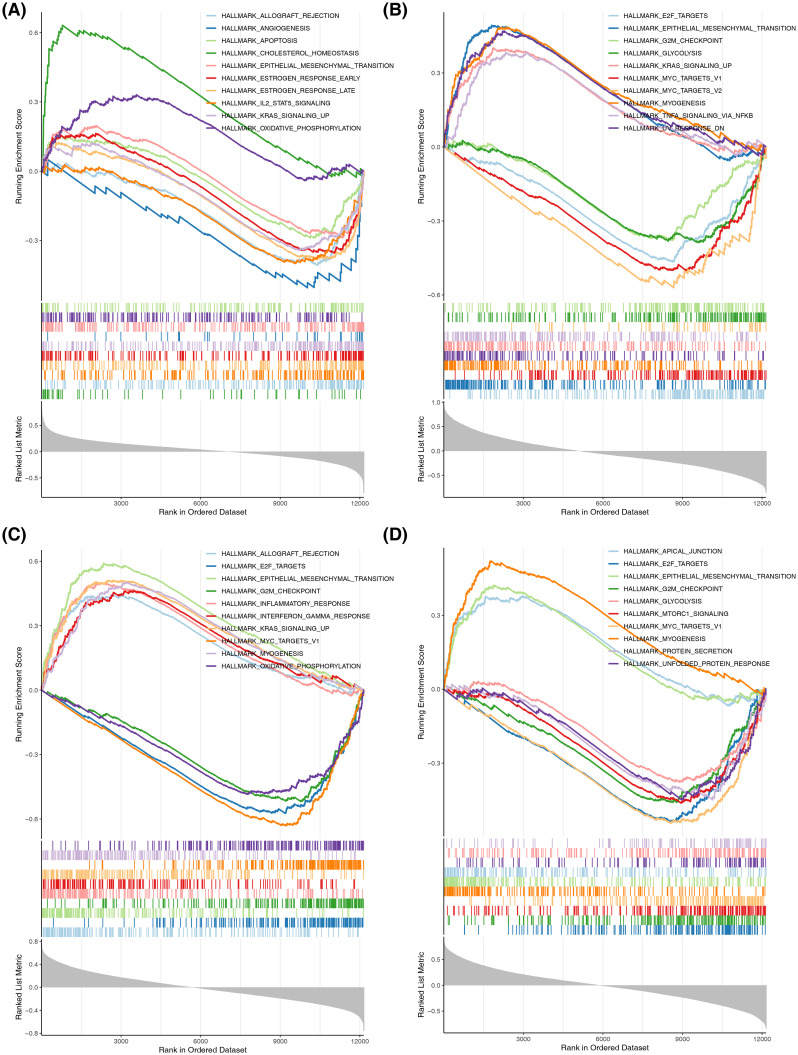
Gene set enrichment analysis (GSEA) results of key OSRG-related signaling pathways. **(A)** The *CYP17A1*-related signaling pathways. **(B)** The *NR3C1*-related signaling pathways. **(C)** The *ENO2*-related signaling pathways. **(D)** The *NGF*-related signaling pathways.

To further determine the OSRG-related functions, GSEA was performed to characterize the potential pathways impacted by the key OSRGs. *CYP17A1, NR3C1, ENO2,* and *NGF* were co-enriched in epithelial-mesenchymal transition; *CYP17A1, NR3C1,* and*ENO2* were co-enriched in *KRAS* signaling; *NR3C1, ENO2,* and *NGF* were co-enriched in E2F targets, G2M checkpoint, MYC targets v1, and myogenesis pathways. These analyses revealed the importance of these OSRG-related pathways in the pathogenesis of EM (**Figures 8A–D**).

### 3.7 Difference in the composition of immune cells and immune-related OSRGs between EC and EU samples

**Figure 9:**
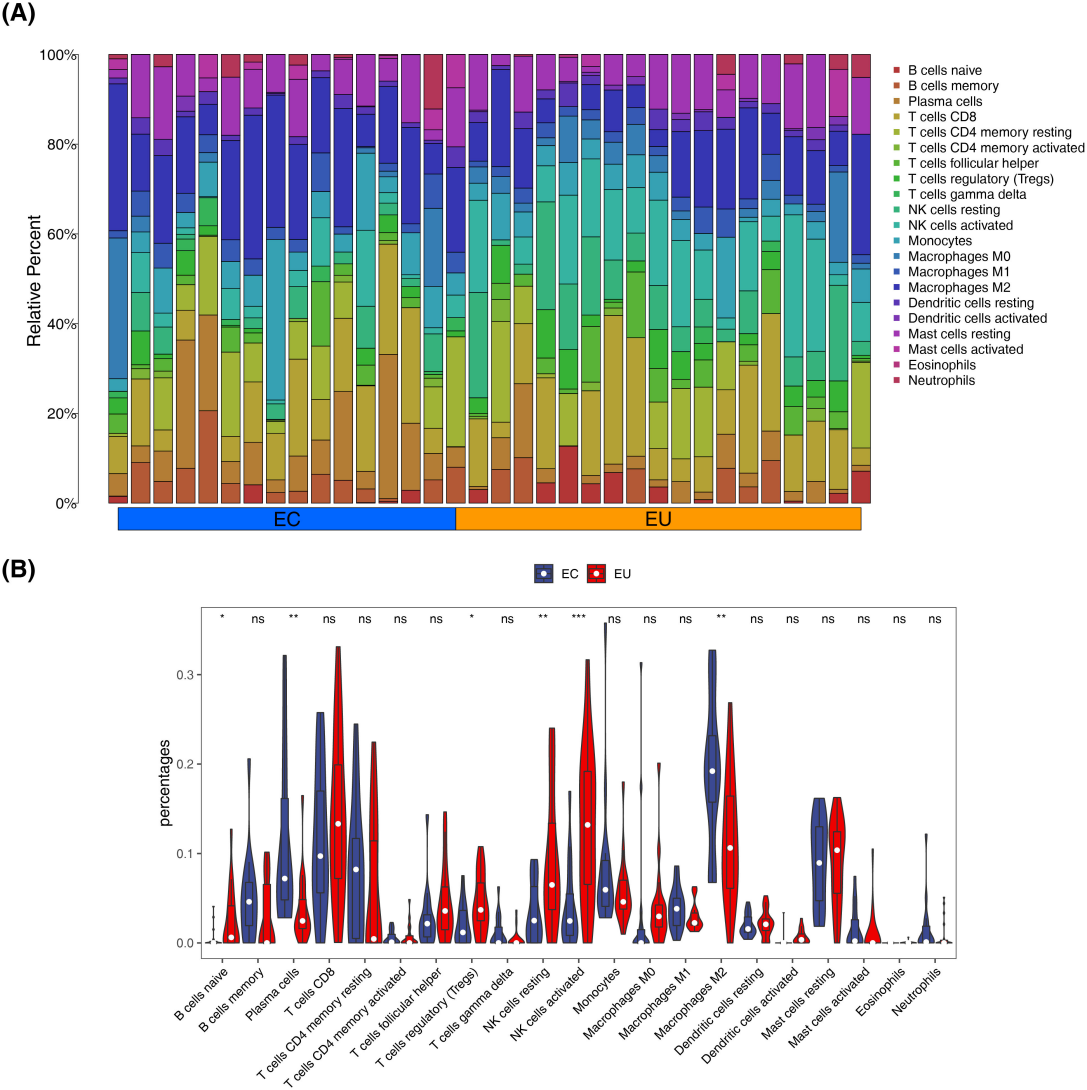
Immune infiltration analysis from EC and EU samples. **(A)** The distribution of 22 distinct immune cell types across each specimen. **(B)** The proportion of six types of immune cells exhibited significant differences between EC and EU samples. *P < 0.05, **P < 0.01, ***P < 0.001, ns: *P* > 0.05.

Based on the combined dataset, we performed an immune infiltration analysis between EC and EU samples to assess the composition of immune cells in the immune microenvironment of EM patients. **Figure 9A** illustrates the proportion of 22 types of immune cells in each sample within the combined dataset. The proportion of six types of immune cells exhibited significant differences between EC and EU samples (*P* < 0.05) (**Figure 9B**). The proportion of plasma cells and M2 macrophages was significantly higher in EC samples (*P* < 0.05). Conversely, naïve B cells, regulatory T cells (Tregs), resting natural killer (NK) cells, and activated NK cells were significantly lower in EC samples compared with EU samples (*P* < 0.05). These results indicated that these immune cells might influence the development of EM.

**Figure 10:**
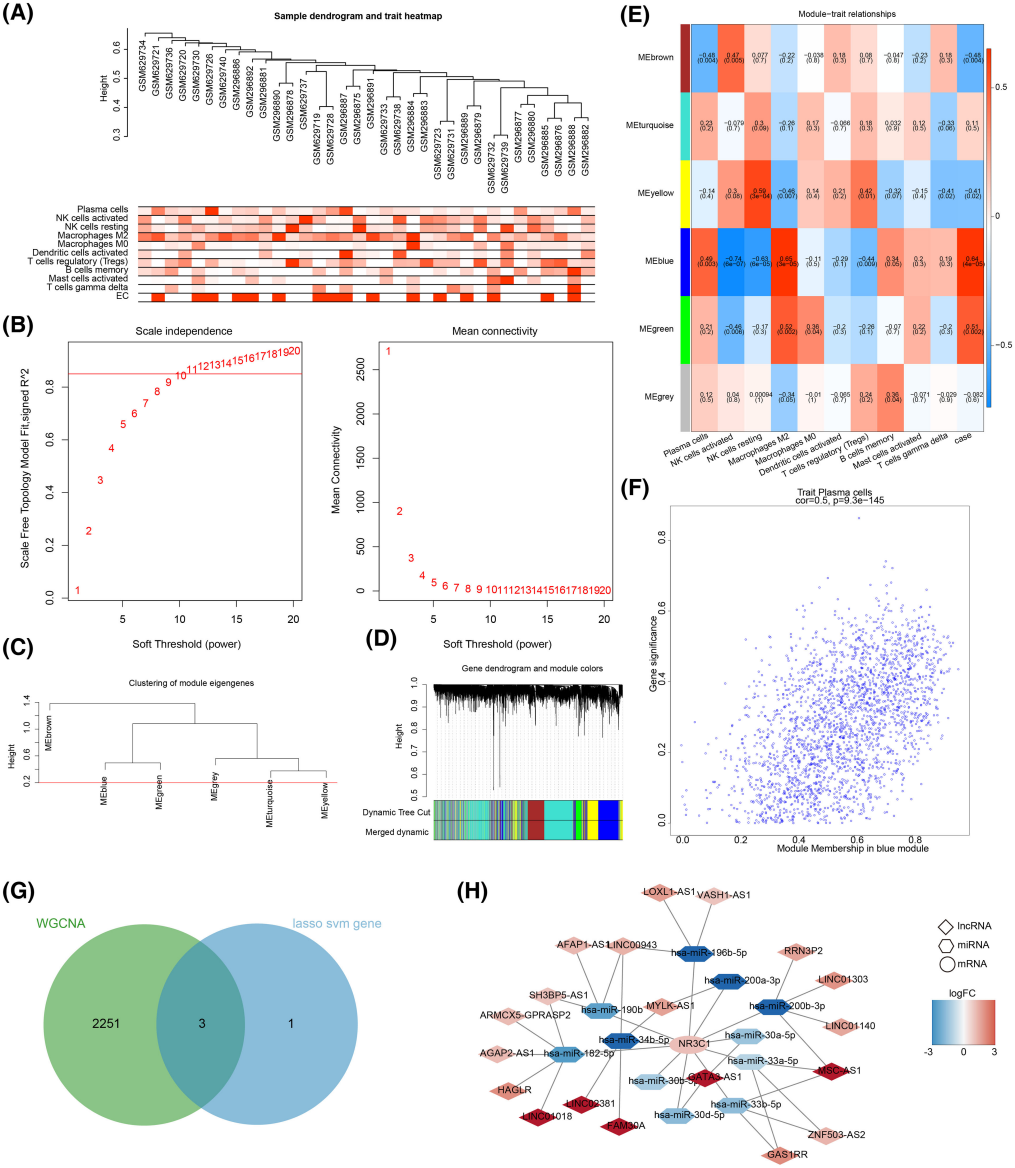
Identification of immune-related module genes via weighted gene co-expression network analysis (WGCNA). **(A)** The results of cluster analysis indicated the absence of outlier samples. **(B)** The optimal soft threshold (β) was finally chosen as 11. **(C, D)** By constructing a co-expression network and setting the minimum number of genes per gene modules to 300, six modules were obtained. **(E, F)** According to the scoring correlation, the MEblue module was taken as a key module with 2,284 genes. **(G)** *CYP17A1, NR3C1*, and *NGF* were acquired by overlapping key OSRGs with immune-related module genes. **(H)** The ceRNA regulation network of *NR3C1*.

To determine if OSRGs were involved in the immune microenvironment of EM pathogenesis, WGCNA was first constructed to identify immune-related module genes. Cluster analysis indicated the absence of outlier samples (**Figure 10A**). The optimal soft threshold (β) was finally chosen as 11 (R^2^ close to 0.85 and mean connectivity close to 0) (**Figure 10B**). By constructing a co-expression network and setting the minimum number of genes per gene modules to 300, six modules were obtained (**Figures 10C, D**). The MEblue module (case correlation = 0.64 and *P* < 0.0001) was taken as a key module with 2,284 genes according to scoring correlation (**Figures 10E, F**). After overlapping key OSRGs with immune-related module genes, three key immune-related OSRGs, including *CYP17A1*, *NR3C1*, and *NGF*, were identified (**Figure 10G**). Moreover, the ceRNA network showed that 20 lncRNAs regulated *NR3C1* through 10 miRNAs, with a total 40 predicted interactions, such as *NR3C1-hsa-miR-30a-5p-GATA3AS1, NR3C1-hsa-miR-196b-5p-LOXL1-AS1*, and *NR3C1-hsa-miR-182-5p-LINC01018* (**Figure 10H**).

## 4 Discussion

EM is a progressive disease associated with severe pain, abdominal bloating, nausea, bowel movements, and infertility. Early diagnosis and treatment of EM are very important, but the diagnosis of EM is often delayed (39–42), which affects the efficient treatment of EM. The occurrence and development of EM have been shown to be associated with OS and immune filtration (43). However, detailed information on the link between OS and EM is limited. Here, we performed a comparative transcriptome analysis between EC and EU samples with extensive bioinformatic analysis and validation of key DE-OSRGs in laboratory study settings. We demonstrated for the first time that several key OSRGs, including *NR3C1, NGF, ENO2,* and *CYP17A1,* are critical in EM pathogenesis. These featured OSRGs may function via multilayered intracellular communications and networks that contribute to EM. Notably, the ROC curve revealed that these four OSRGs displayed commendable diagnostic performance.

*CYP17A1,* which is located on chromosome 10q24.3 with 8 exons and 7 introns, encodes a membrane-bound bifunctional monooxygenase that belongs to the CYP450 enzyme superfamily and plays a key role in the androgen and estrogen metabolism pathway (44, 45). CYP17A1 is a key enzyme in steroid hormone synthesis that synthesizes hormones such as progesterone, mineralocorticoids, glucocorticoids, androgens, and estrogen; these hormones can affect the activity and function of immune cells, thereby affecting the inflammatory response. Polymorphisms of the *CYP17A1* gene might affect the metabolism of estrogen and contribute to the onset of EM (46, 47). Estrogen plays a crucial role in the occurrence and development of EM, and abnormal expression of CYP17A1 may lead to elevated estrogen levels, thereby promoting the growth of ectopic endometrial cells (48, 49). Therefore, CYP17A1 can serve as a potential therapeutic target to regulate the progression of EM by modulating its activity. ENO2 is an intracellular enzyme involved in glycolysis that catalyzes the dehydration of 2-phospho-D-glycerol esters to phosphoenolpyruvate. This conversion process can promote the conversion of glucose to pyruvate, a precursor that promotes the production of NADH molecules and high-energy ATP (50, 51). Many enzymes involved in the glycolytic pathway play important roles in biological processes, tumor progression, and disease occurrence (52–54). Research has shown that *ENO2* is a reliable biomarker for diagnosing cancer with great potential for use in various tumors such as melanoma and seminoma (55). The mutation of ENO2 significantly reduces the phosphorylation level of PKM2, while PAK5mediated PKM2 phosphorylation can increase its enzyme activity, promote glucose metabolism, and support the survival and proliferation of EM cells in hypoxic environments (56, 57). Therefore, mutations in ENO2 may indirectly affect the metabolic status of EM by affecting the phosphorylation of PKM2. *NR3C1* is a glucocorticoid receptor gene that participates in glucocorticoid signal transduction and plays a crucial role in immune and inflammatory responses. Corticosteroids are important regulatory factors in immune and inflammatory responses that can inhibit inflammatory responses and regulate the activity and function of immune cells. Mutations in the *NR3C1* gene can lead to glucocorticoid resistance, affecting immune and inflammatory responses (58). Abnormalities in this function may lead to increased expression of inflammatory factors such as TNF-α, which can induce angiogenesis, promote endometrial cell proliferation and intercellular adhesion, and drive the formation and development of ectopic lesions (59, 60). Anaf et al. found that in the most symptomatic deep glandular nodules of EM, significant *NGF* expression and invasion of the perineum and neuro-endometrium were observed (61). Research has found that the nerve fiber density and *NGF* expression level are much higher in deep infiltrating EM than in superficial peritoneal EM (62). High expression of NGF can promote the regeneration and proliferation of nerve fibers, thereby increasing the sensitivity of nociceptors and exacerbating pain (63). These studies indicate that NGF is closely related to EM and its associated pelvic pain (64, 65). In summary, CYP17A1, ENO2, NR3C1, and NGF were associated with EM from different perspectives, such as hormone metabolism, glycolysis metabolism, inflammatory response, and neural correlation, which provided important evidence for analyzing the pathogenesis and exploring new management methods of EM.

We conducted validation studies and found that the mRNA and protein levels of *NR3C1, NGF,* and *ENO2* were significantly elevated in EC compared with EU, whereas the levels of *CYP17A1* was the opposite. This was consistent with the patterns observed in public databases used for our study. Based on these findings, we hypothesized that ENO2 could facilitate the conversion of glucose to pyruvate, thereby supplying the necessary energy for the adhesion and proliferation of ectopic endometrial cells, leading to the emergence and progression of EM. *NR3C1* may lead to glucocorticoid resistance, impacting the body’s immunity and triggering inflammatory responses, which could precipitate EM. Moreover, CYP17A’s influence on estrogen metabolism might contribute to the progression of EM, while NGF could exacerbate pelvic pain in EM through OS. No studies on the association of *ENO2* and *NR3C1* with EM have been published. Our investigation is the first to link these genes with EM, thereby introducing a novel perspective for future research. Additional studies are required to confirm the involvement of these signature genes in the etiology of EM.

Enrichment analysis shows that *CYP17A1, NR3C1, ENO2,* and *NGF* are co-enriched in the epithelial-mesenchymal transition pathway (EMT), a key mechanism in morphogenesis and organogenesis (66). Numerous studies have shown that EMT is closely related to wound healing, fibrosis, tissue regeneration, inflammation, and cancer metastasis (67–69). EMT is intrinsically tied to EM development and formation (70). During this process, the epithelial cells of the endometrium are transformed into stromal cells through EMT, and these cells may then spread to the outside of the uterus through the blood or lymphatic system and form endometriotic lesions in other areas (71). Moreover, EMT appears to contribute to the invasion and metastasis of EM (72). *CYP17A1,* in addition to the EMT pathway, *NR3C1* and *ENO2* are co-enriched in the *KRAS* signaling pathway. EM is characterized by the extraneous growth of endometrial cells, which, during ectopic expansion, may distress surrounding tissues and organs, resulting in pain and infertility, among other symptoms. Studies have indicated that abnormal activation of the KRAS signaling pathway may play a crucial role in this process (72–74). KRAS activation accelerates endometrial cell proliferation and growth, expediting the formation and progression of ectopic lesions (75). KRAS activation may enhance cell mobility, facilitating the spread of endometrial cells beyond the uterus (76). Hence, investigating the role of the *KRAS* signaling pathway in EM could yield new therapeutic targets and strategies. Finally, *NR3C1, ENO2,* and *NGF* are co-enriched in G2M checkpoint, E2F targets, MYC targets V1, and myogenesis pathways. Research has found that G2M checkpoints play a vital role in the pathogenesis of EM. When cells are subjected to certain stress stimuli, such as DNA damage or abnormal cell division, G2M checkpoints are activated to prevent damaged cells from entering the division cycle (77). In EM, damaged endometrial cells may avoid excessive proliferation and carcinogenesis by activating G2M checkpoints (78). There is no evidence to suggest a direct relationship between EM with E2F targets, MYC targets V1, and myogenesis pathways. Any new research or discoveries in these areas would warrant further exploration.

Abnormal immune regulation is closely related to the pathogenesis of EM, manifested by weakened immune surveillance function and cytotoxic effects of immune killing cells, which cannot effectively clear EC (79, 80). In our investigation, we used CIBERSORT to assess the immune cell infiltration present in EC and EU specimens. The results indicated the involvement of various immune cell subsets in the onset and development of EM. We observed an increased presence of plasma cells and M2 macrophages, alongside a reduced infiltration of immature B cells, Tregs, resting NK cells, and activated NK cells. These fluctuations are potentially implicated in the emergence and progression of EM. As EM is an immune-related condition, the invasive and adhesive properties of endometrial cells are amplified because of the quantity and functionality of diverse immune cells within the peritoneal milieu. This includes plasma cells, M2 macrophages, NK cells, macrophages, dendritic cells, mast cells, and T cells. These cells facilitate angiogenesis and support the formation and persistence of ectopic growths. In EM, NGF may affect the immune response by regulating the differentiation and survival of T and B cells; CYP17A1 can regulate estrogen levels to promote the proliferation of Tregs, thereby inhibiting the TGF-β signaling pathway or reducing the number of Tregs, which may help enhance the immune system’s ability to clear ectopic lesions (81–83). They also aid in the implantation of EC cells, which may be transported back to the pelvic and peritoneal cavities with the menstrual blood, thereby circumventing immune surveillance (84). Endometrioid tissue may spread beyond its endometrial position during the menstrual cycle and implant in other locations; the most common is the formation of lesions by implanting pelvic structures (85). This damage attracts natural killer cells, macrophages, and cytotoxic T cells, and the immune response seems insufficient to clear them (86). The secretion of chemokines and cytokines in the peritoneal cavity can be increased by activating the inflammatory response, creating a microenvironment that promotes local angiogenesis and disrupts endometrial apoptosis, contributing to the development of EC tissue (87). This evidence combined with our current research findings underscores that the infiltration of various immune cells is pivotal in the context of EM and, as such, should be the focal point of future investigations.

The ceRNA network we developed showed that 20 lncRNAs contributed to the regulation of *NR3C1* via 10 distinct miRNAs, forming a total of 40 interaction pairs. NR3C1 modulates the activity of steroid hormone receptors and plays a consequential role in EM, exhibiting a close association with the risk and severity of EM symptoms (88). miR-200a and miR-200b belong to the miR-200 family, which are the upstream targets of *NR3C1*. The miR-200 family regulates the transcription factor network involved in EMT, which is considered a key event in the progression of EM (65). Studies have shown that miR-200a and miR-200b have significant links to the development of EM, regulating the proliferation, apoptosis, and invasion of ectopic endometrial cells (89–93). Consistent with our results, miRNAs of the miR-200 family were downregulated in ectopic endometrium compared with the *in-situ* endometrium, and lower levels of the miR-200 family were believed to participate in the pathogenesis of EM by stimulating cell movement and inducing EMT processes (94–97). The recently identified *LncRNA-MSC-AS1* has been associated with the genesis and progression of diverse cancers, including its role in promoting osteogenic differentiation in bone marrow–derived mesenchymal stem cells (98). *LncRNA MSC-AS1* may also worsen the progression of nasopharyngeal carcinoma through upregulation of *NR4A2* via sequestration of *miR-524-5p* (99). A study by Hu and colleagues indicated that *MSC-AS1* modulates the proliferation and migration of kidney renal clear cell carcinoma by acting on the *miR3924/WNT5A/β-catenin* pathways (91). Additionally, a study by Chenglei and colleagues revealed a significant link between lncRNA *MSC-AS1* and EM (100).

Bioinformatics results indicated that *LncRNA MSC-AS1/NR3C1* was increased while *miR-200b-8p* was reduced. Based on the ceRNA mechanism, we speculate that *lncRNA MSC-AS1* enhances the level of *NR3C1* by absorbing *miR-200b-8p*, thereby exacerbating OS during the progression of EM. However, more studies and large-scale randomized controlled clinical trials are required to validate this vital OS-related axis in EM. Multiple reports have suggested a strong correlation between OS and the pathophysiology of EM (13, 101). The apoptotic endometrial tissue, macrophages, and red blood cells produced by retrograde menstruation promote the production of ROS (102). The increase in ROS levels induces a general inflammatory response, which is beneficial for the adhesion and growth ofendometrial cells detached from the peritoneal cavity (13). Research on the potential use of OS markers as diagnostic tests for EM is still in its early stages (103–105).

It is worth noting that there was no differential expression of eosinophils between EC and EA samples in our study. Previous studies have shown a close relationship between eosinophils and oxidative stress, and an increase in eosinophils is often associated with allergic reactions, autoimmune diseases, or certain tumors. However, the research on eosinophils in EM has not been reported (106). Our study suggests that eosinophils may not be directly associated with EM. In addition, there is currently no direct evidence about the role of CYP*17A1*, *NR3C1*, *ENO2*, and *NGF* genes, and their related miRNAs in regulating eosinophils. Although this study showed a decline in inactive B cells, regulatory B cells, and NK cells in EC, there is no direct evidence to suggest that the reduction of those cells would lead to an increase in eosinophils. The changes in these immune cells may reflect alterations in the immune microenvironment of EM patients rather than simply caused by changes in the number of specific immune cells. Although eosinophils play an important role in immune responses, the molecular mechanism underlying aberrant eosinophils contributing to EM is unclear, and future research is needed to further explore the specific mechanisms of eosinophils in EM.

Our research has several limitations. Firstly, the diagnostic performance of machine learning models has not been externally validated, which limits their widespread application in a wider population. Secondly, due to the small sample size, the generalizability of research results may be affected. Therefore, larger-scale clinical and basic research is needed to confirm the featured OSRGs we identified. In addition, although the CIBERSORT algorithm performs relatively low in evaluating bias compared to other algorithms, it still has inherent limitations in overestimating or underestimating cell subsets. Therefore, future research can optimize model performance by adjusting algorithm parameters and attempting to use more complex algorithms, such as deep learning, to further improve prediction accuracy. In order to enhance the reliability and application breadth of the model, more clinical data should be introduced for validation in the future. Moreover, increasing the sample size, especially including patients with EM from different types and stages, can improve the representativeness and application value of research results. Furthermore, expanded validation of miRNA, lncRNA, and other DEGs at RNA or protein can provide more solid experimental support for our findings. These efforts will provide a more comprehensive theoretical basis and practical guidance for the precise diagnosis and treatment of EM in the future.

In conclusion, we identified several featured OSRGs, including *CYP17A1, ENO2, NGF,* and *NR3C1,* critical in EM pathogenesis. Several types of immune cells, including plasma cells, M2 macrophages, Tregs, resting NK cells, naive B cells and activated NK cells are closely related to the pathogenesis of EM. The functional enrichment analysis results of this study also showed that the screened immune-related OS genes were mainly enriched in OS and immune-related pathways. Therefore, based on these findings, early diagnosis of EM can improve accuracy and timeliness by detecting the expression levels of these biomarkers. The development of drugs targeting these OSRGs was expected to alleviate EM symptoms by reducing oxidative stress, providing new targets for treatment. In addition, the infiltration pattern of immune cells can serve as a biomarker for evaluating the immune microenvironment, helping to determine the severity and progression of diseases. Finally, combining hormone therapy, immune regulation, and antioxidant therapy to develop a multi-target combination therapy strategy will help provide a more comprehensive intervention plan, thereby improving the clinical prognosis of patients. Overall, this study not only provides new insights into the pathogenesis, but also offers potential directions for clinical diagnosis and treatment of EM.

## Data Availability

The original contributions presented in the study are included in the article/**Supplementary Material**. Further inquiries can be directed to the corresponding author.
