# Supplementary figures and images for: Identification and analysis of oxidative stress-related genes in endometriosis
Source: Front Immunol. 2025 Mar 7;16:1515490. doi: 10.3389/fimmu.2025.1515490 (PMC11925871; doi:10.3389/fimmu.2025.1515490)

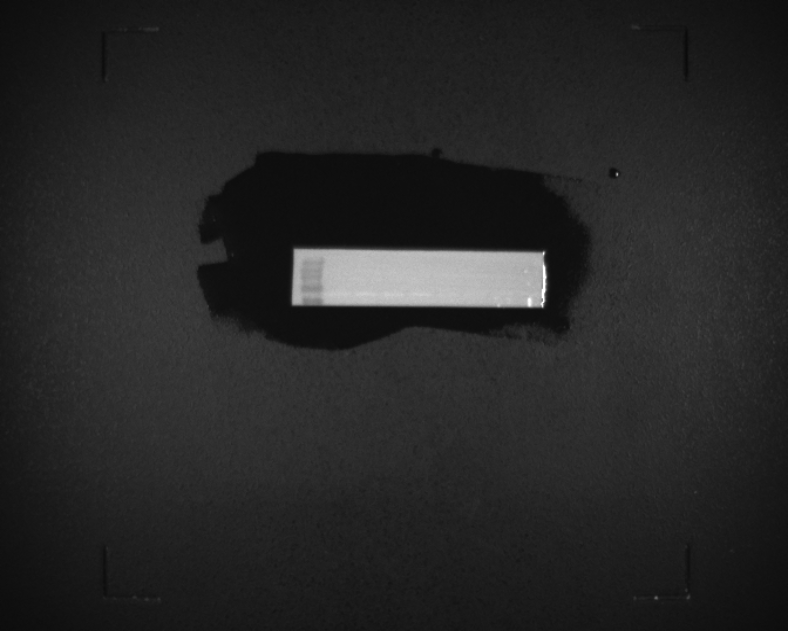

Supplement: Supplementary file 2 [file DataSheet2.zip › The original image files for the blots-2/NR3C1/Sage_maker_20240125_105729.tif]

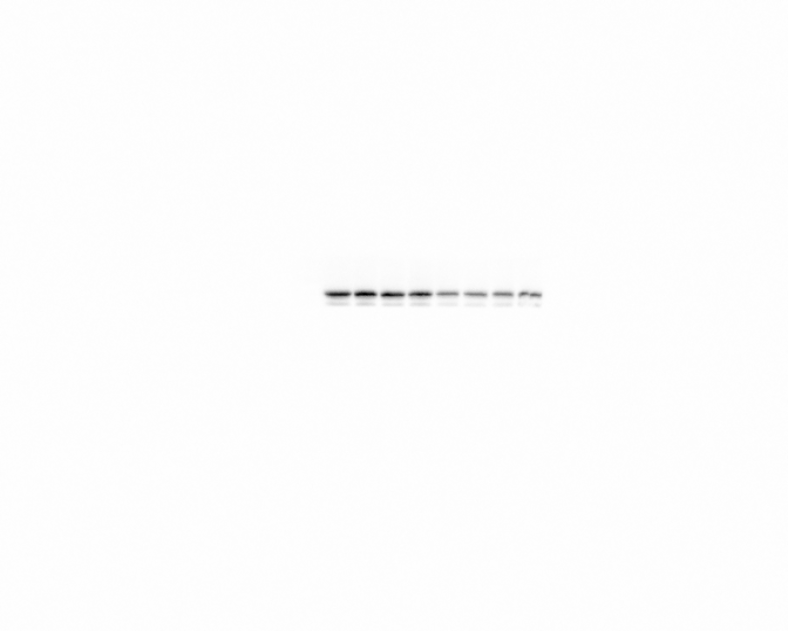

Supplement: Supplementary file 2 [file DataSheet2.zip › The original image files for the blots-2/NR3C1/Sage_原始图_20240125_105729_200ms.tif]

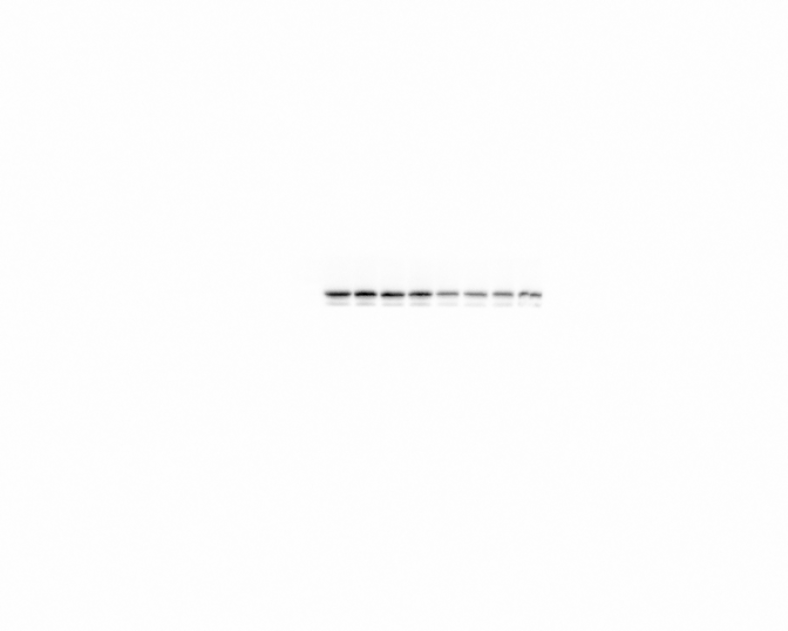

Supplement: Supplementary file 2 [file DataSheet2.zip › The original image files for the blots-2/NR3C1/Sage_原始图_20240125_105729_200ms_wps图片_1.png]

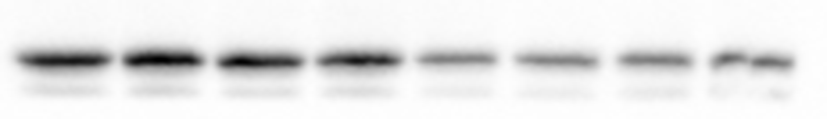

Supplement: Supplementary file 2 [file DataSheet2.zip › The original image files for the blots-2/NR3C1/Sage_原始图_20240125_105729_200ms_wps图片_1.tif]

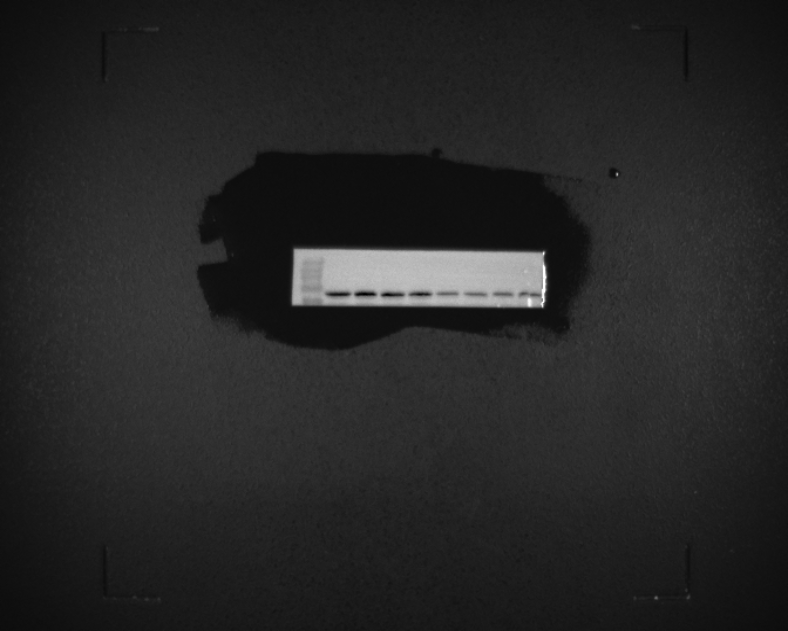

Supplement: Supplementary file 2 [file DataSheet2.zip › The original image files for the blots-2/NR3C1/Sage_合成图_20240125_105729_200ms.tif]

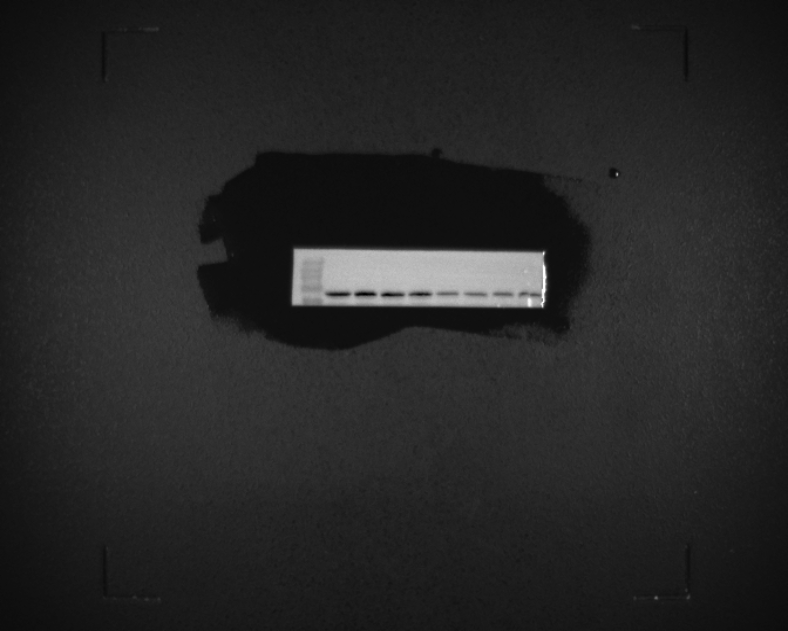

Supplement: Supplementary file 2 [file DataSheet2.zip › The original image files for the blots-2/NR3C1/Sage_合成图_20240125_105729_200ms_wps图片_1.png]

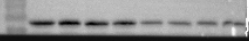

Supplement: Supplementary file 2 [file DataSheet2.zip › The original image files for the blots-2/NR3C1/Sage_合成图_20240125_105729_200ms_wps图片_1.tif]

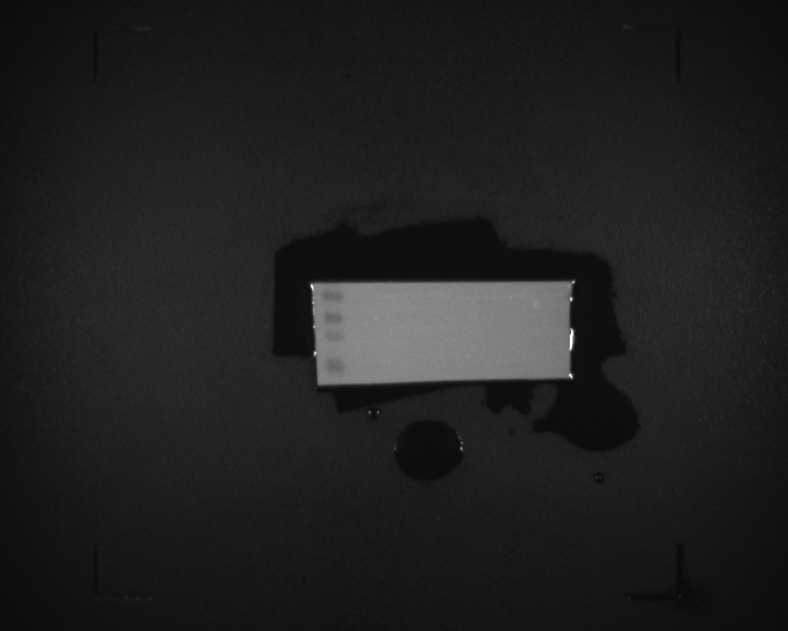

Supplement: Supplementary file 2 [file DataSheet2.zip › The original image files for the blots-2/β-actin/Sage_maker_20240125_112614.tif]

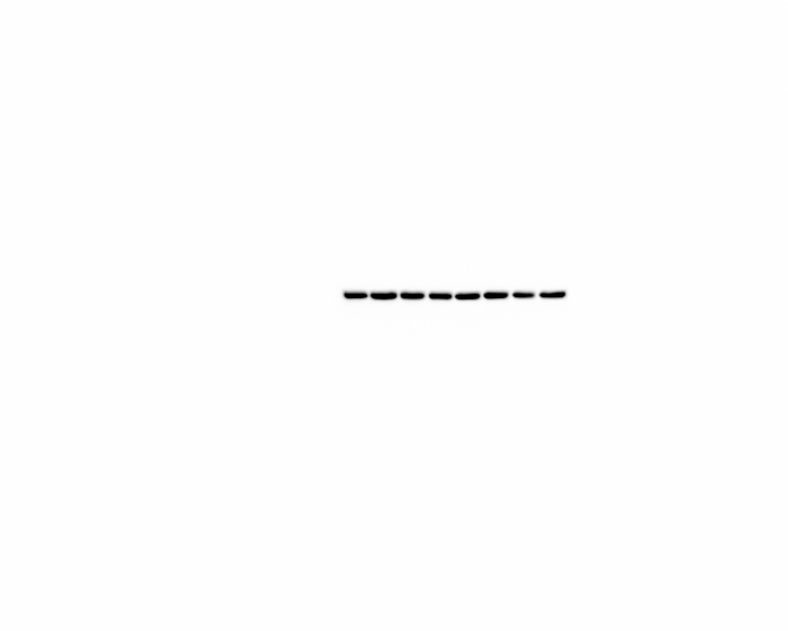

Supplement: Supplementary file 2 [file DataSheet2.zip › The original image files for the blots-2/β-actin/Sage_原始图_20240125_112614_2s50ms.tif]

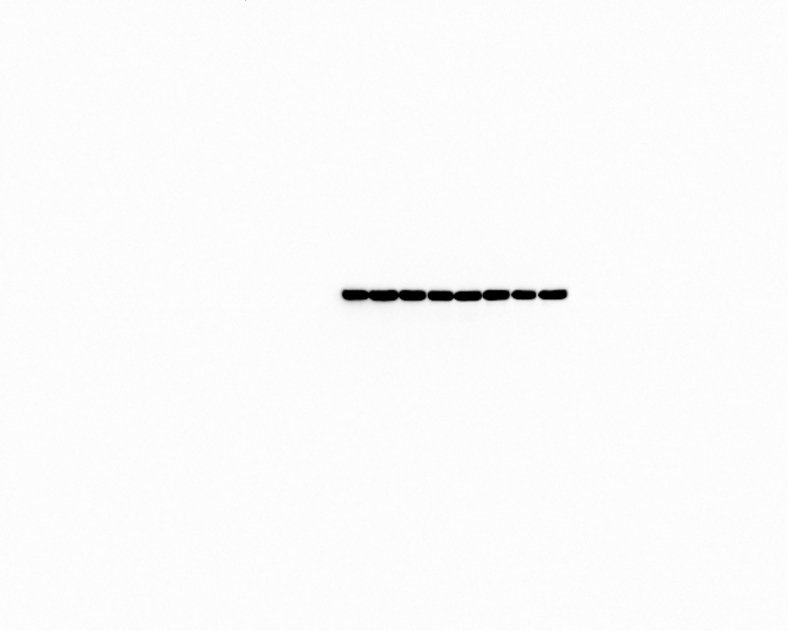

Supplement: Supplementary file 2 [file DataSheet2.zip › The original image files for the blots-2/β-actin/Sage_原始图_20240125_112614_2s50ms_wps图片_5.png]

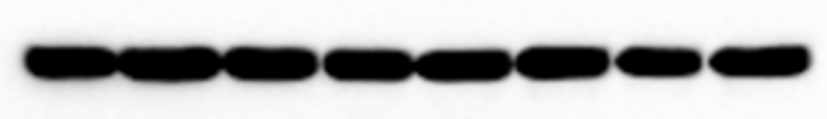

Supplement: Supplementary file 2 [file DataSheet2.zip › The original image files for the blots-2/β-actin/Sage_原始图_20240125_112614_2s50ms_wps图片_5.tif]

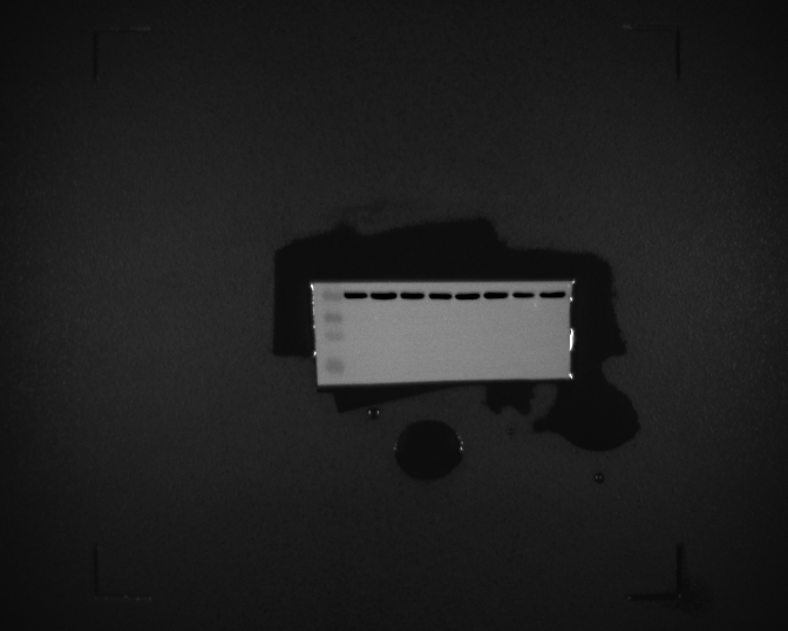

Supplement: Supplementary file 2 [file DataSheet2.zip › The original image files for the blots-2/β-actin/Sage_合成图_20240125_112614_2s50ms.tif]

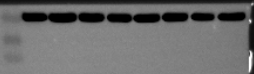

Supplement: Supplementary file 2 [file DataSheet2.zip › The original image files for the blots-2/β-actin/Sage_合成图_20240125_112614_2s50ms_wps图片_3.png]

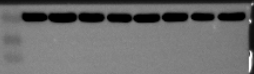

Supplement: Supplementary file 2 [file DataSheet2.zip › The original image files for the blots-2/β-actin/Sage_合成图_20240125_112614_2s50ms_wps图片_3.tif]

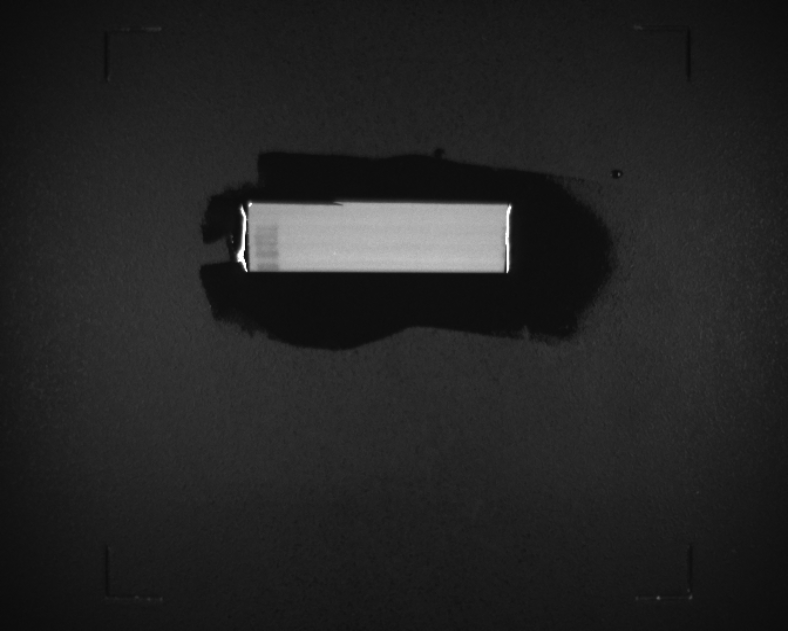

Supplement: Supplementary file 3 [file DataSheet3.zip › The original image files for the blots-1/CYP17A1/Sage_maker_20240125_105648.tif]

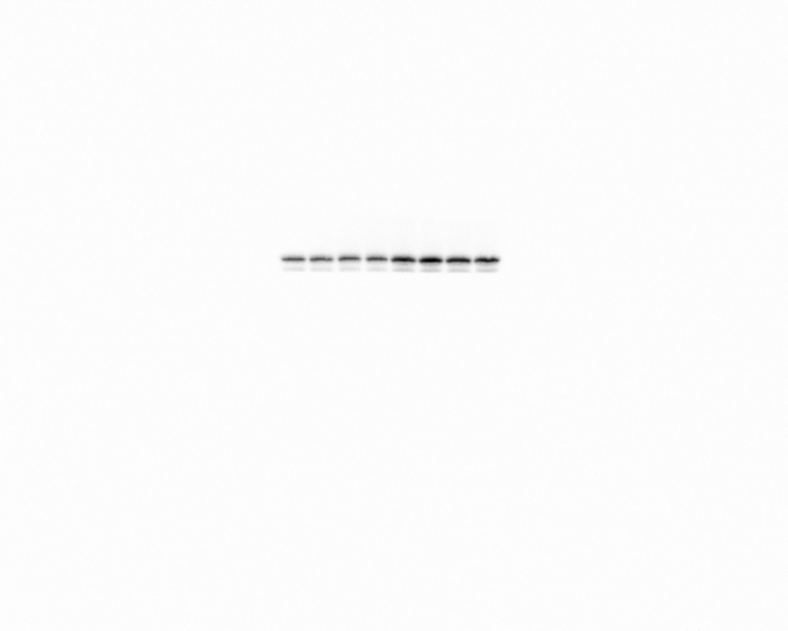

Supplement: Supplementary file 3 [file DataSheet3.zip › The original image files for the blots-1/CYP17A1/Sage_原始图_20240125_105648_200ms.tif]

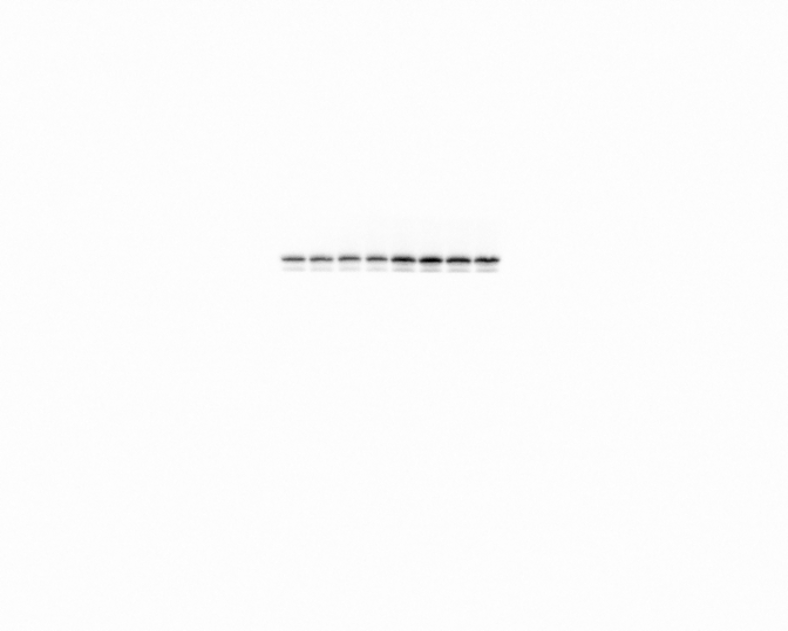

Supplement: Supplementary file 3 [file DataSheet3.zip › The original image files for the blots-1/CYP17A1/Sage_原始图_20240125_105648_200ms_wps图片_1.png]

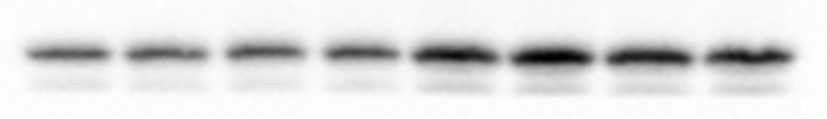

Supplement: Supplementary file 3 [file DataSheet3.zip › The original image files for the blots-1/CYP17A1/Sage_原始图_20240125_105648_200ms_wps图片_1.tif]

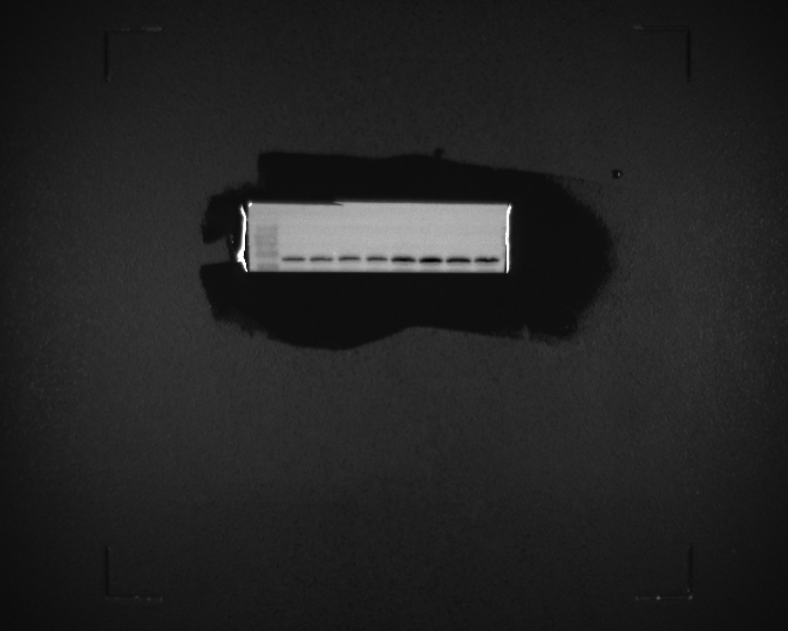

Supplement: Supplementary file 3 [file DataSheet3.zip › The original image files for the blots-1/CYP17A1/Sage_合成图_20240125_105648_200ms.tif]

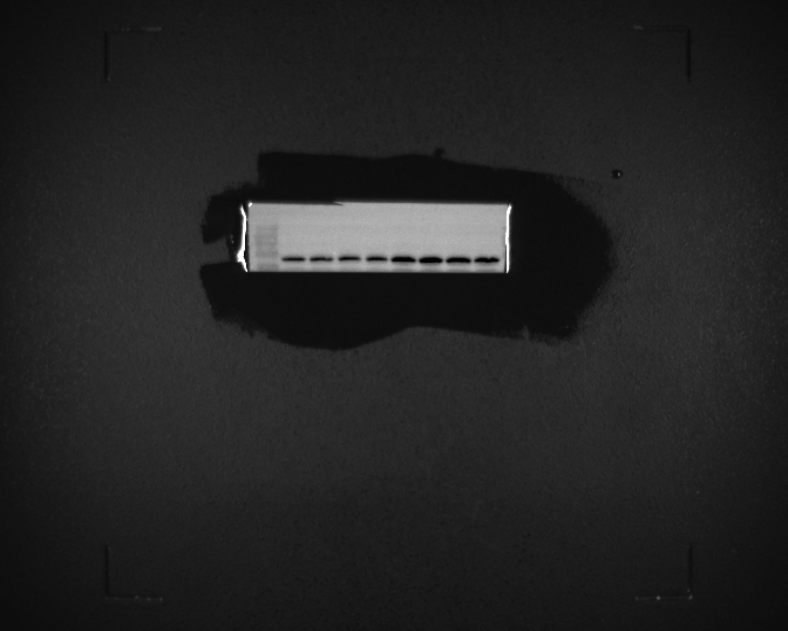

Supplement: Supplementary file 3 [file DataSheet3.zip › The original image files for the blots-1/CYP17A1/Sage_合成图_20240125_105648_200ms_wps图片_3.png]

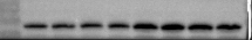

Supplement: Supplementary file 3 [file DataSheet3.zip › The original image files for the blots-1/CYP17A1/Sage_合成图_20240125_105648_200ms_wps图片_3.tif]

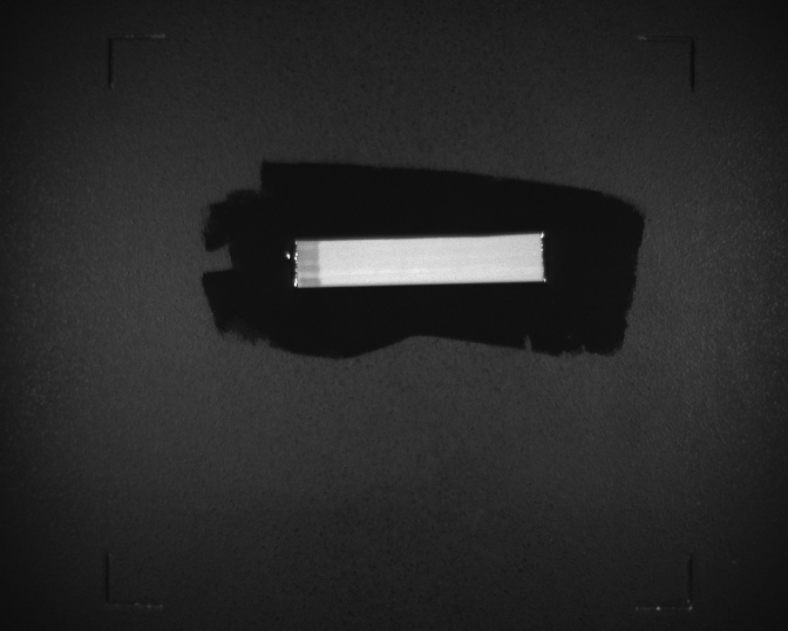

Supplement: Supplementary file 3 [file DataSheet3.zip › The original image files for the blots-1/ENO2/Sage_maker_20240125_105427.tif]

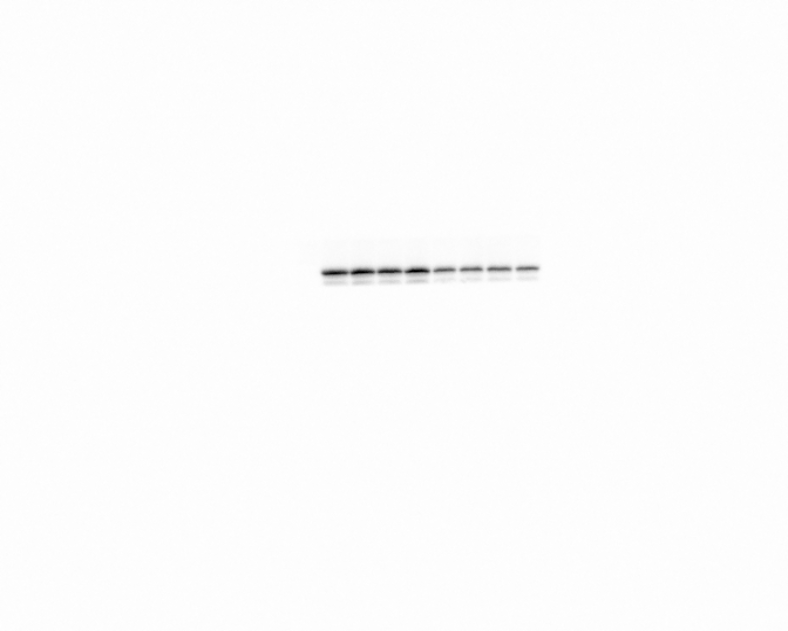

Supplement: Supplementary file 3 [file DataSheet3.zip › The original image files for the blots-1/ENO2/Sage_原始图_20240125_105427_200ms.tif]

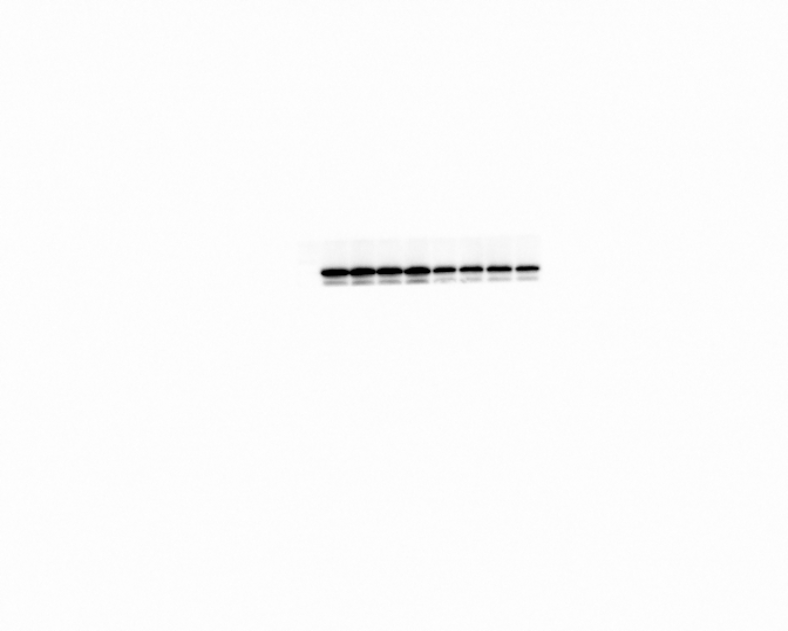

Supplement: Supplementary file 3 [file DataSheet3.zip › The original image files for the blots-1/ENO2/Sage_原始图_20240125_105427_200ms_wps图片_4.png]

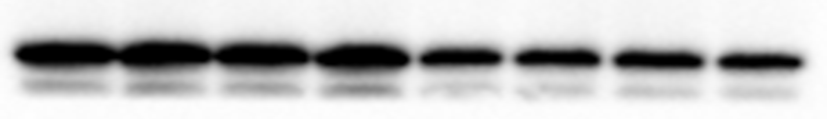

Supplement: Supplementary file 3 [file DataSheet3.zip › The original image files for the blots-1/ENO2/Sage_原始图_20240125_105427_200ms_wps图片_4.tif]

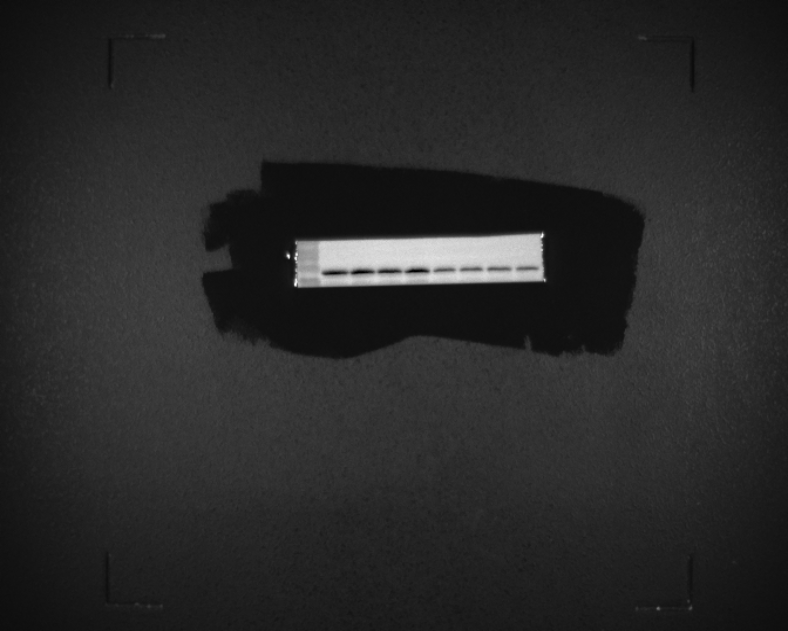

Supplement: Supplementary file 3 [file DataSheet3.zip › The original image files for the blots-1/ENO2/Sage_合成图_20240125_105427_200ms.tif]

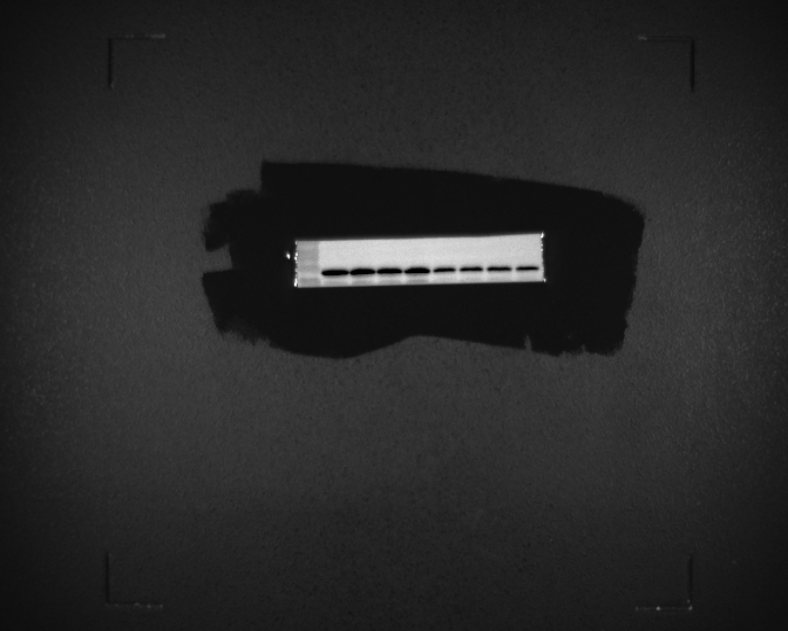

Supplement: Supplementary file 3 [file DataSheet3.zip › The original image files for the blots-1/ENO2/Sage_合成图_20240125_105427_200ms_wps图片_3.png]

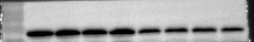

Supplement: Supplementary file 3 [file DataSheet3.zip › The original image files for the blots-1/ENO2/Sage_合成图_20240125_105427_200ms_wps图片_3.tif]

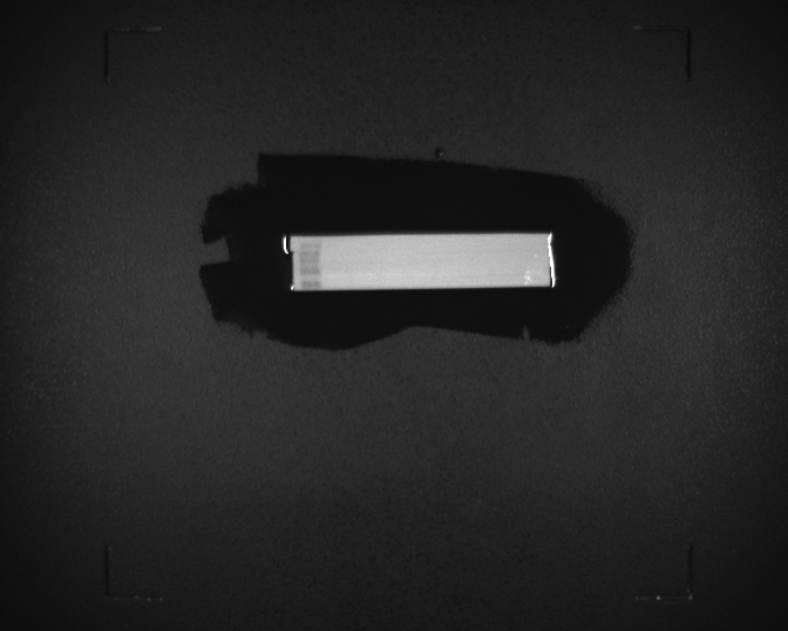

Supplement: Supplementary file 4 [file DataSheet4.zip › The original image files for the blots-3/NGF/Sage_maker_20240125_105602.tif]

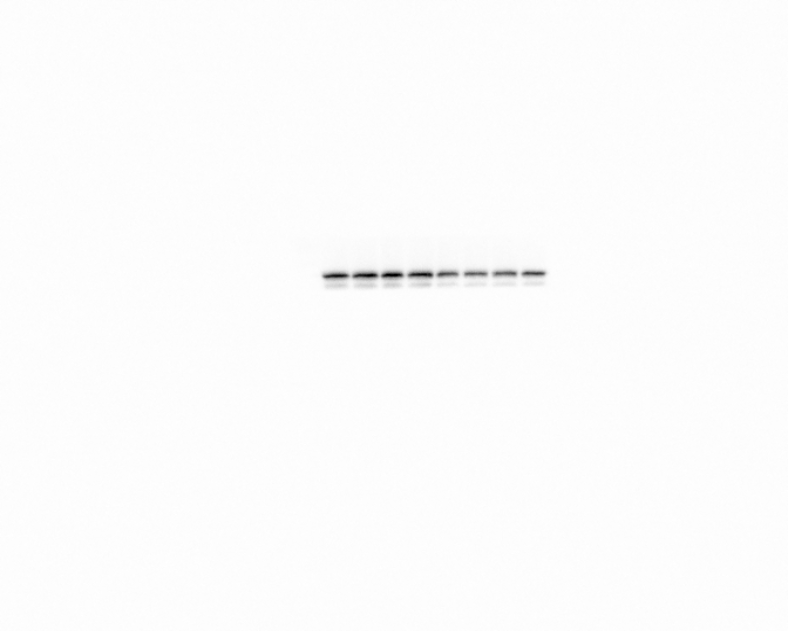

Supplement: Supplementary file 4 [file DataSheet4.zip › The original image files for the blots-3/NGF/Sage_原始图_20240125_105602_200ms.tif]

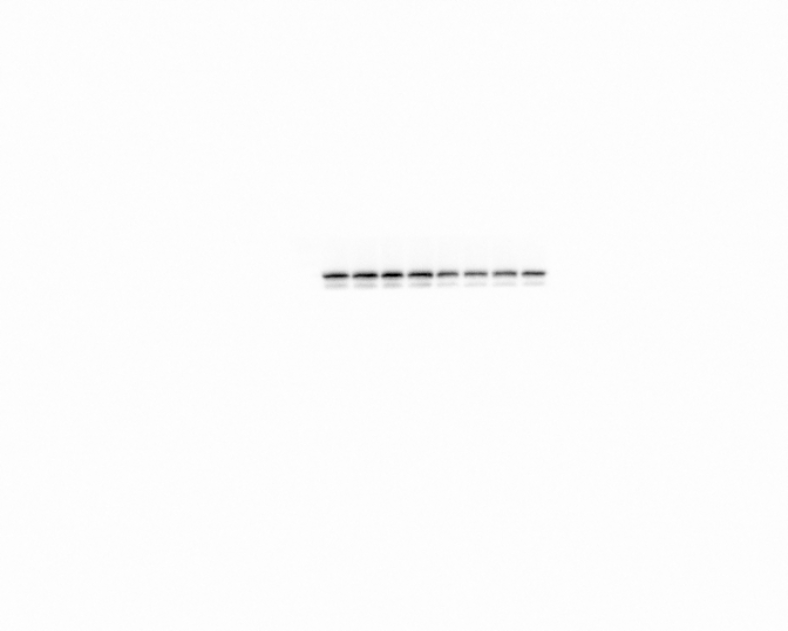

Supplement: Supplementary file 4 [file DataSheet4.zip › The original image files for the blots-3/NGF/Sage_原始图_20240125_105602_200ms_wps图片_1.png]

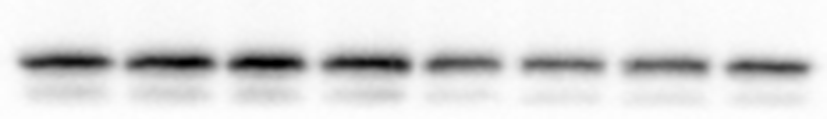

Supplement: Supplementary file 4 [file DataSheet4.zip › The original image files for the blots-3/NGF/Sage_原始图_20240125_105602_200ms_wps图片_1.tif]

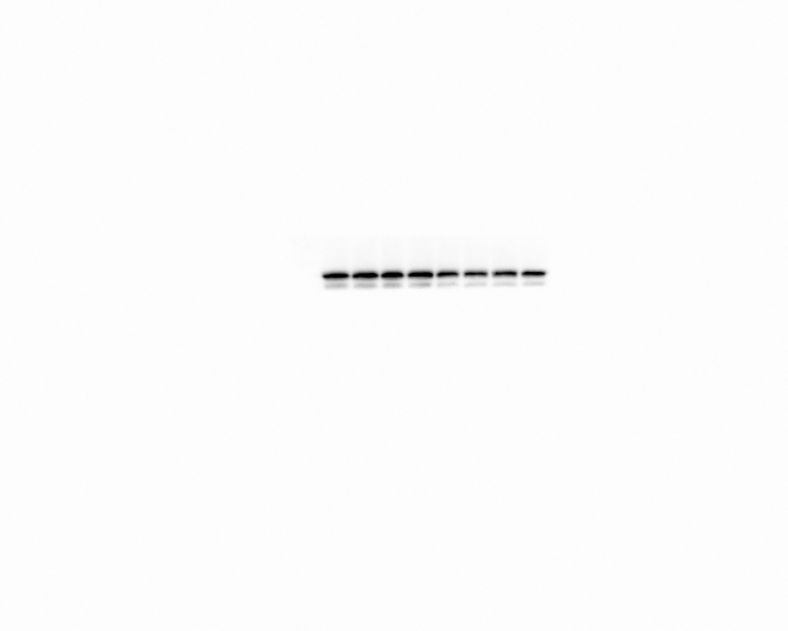

Supplement: Supplementary file 4 [file DataSheet4.zip › The original image files for the blots-3/NGF/Sage_原始图_20240125_105602_200ms_wps图片_3.png]

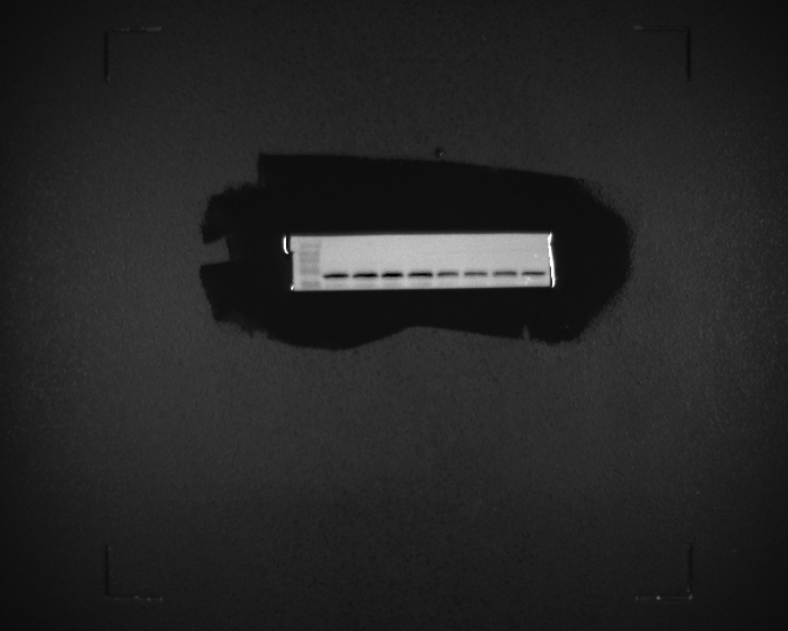

Supplement: Supplementary file 4 [file DataSheet4.zip › The original image files for the blots-3/NGF/Sage_合成图_20240125_105602_200ms.tif]

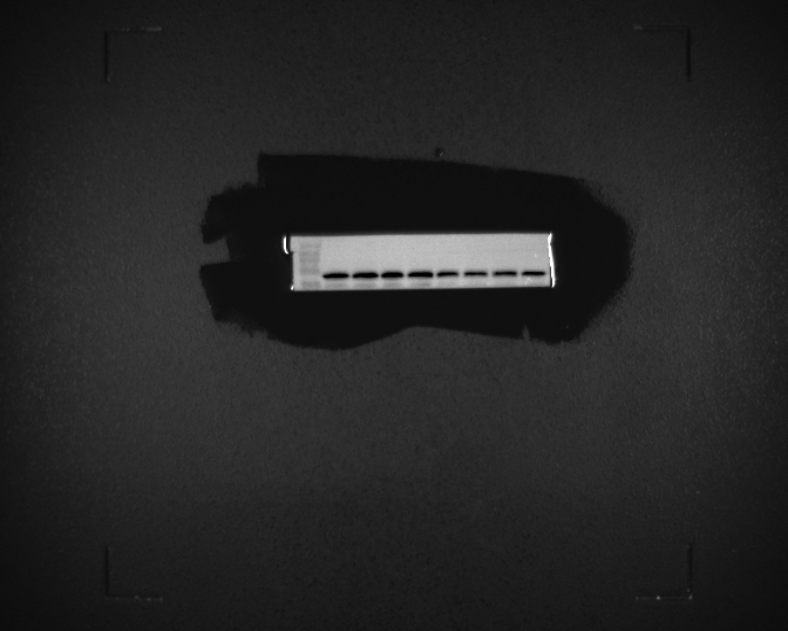

Supplement: Supplementary file 4 [file DataSheet4.zip › The original image files for the blots-3/NGF/Sage_合成图_20240125_105602_200ms_wps图片_3.png]

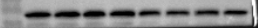

Supplement: Supplementary file 4 [file DataSheet4.zip › The original image files for the blots-3/NGF/Sage_合成图_20240125_105602_200ms_wps图片_3.tif]
